# Effect of Monomer Polarity on Polymer Dynamics, Glass Transition, and Ionic Conductivity of Polyether Electrolytes

**DOI:** 10.1021/acs.macromol.5c02520

**Published:** 2026-01-07

**Authors:** Soma Ahmadi, Danielle DeJonge, Niloofar Safaie, Shaylynn Crum-Dacon, Robert C. Ferrier, Shiwang Cheng

**Affiliations:** Department of Chemical Engineering and Materials Science, 3078 Michigan State University, East Lansing, Michigan 48824, United States

## Abstract

Combining X-ray scattering, Fourier transform infrared spectroscopy (FT-IR), broadband dielectric spectroscopy (BDS), rheology, and differential scanning calorimetry (DSC), we investigate the ion solvation structures, the polymer dynamics, glass transition, and ionic conductivity of poly­(butylene oxide) (PBO, static dielectric constant $\varepsilon_{s}^{\mathrm{PBO}}\approx 4$ )/Lithium bis­(trifluoromethanesulfonyl)­imide (LiTFSI), poly­(propylene oxide) (PPO, $\varepsilon_{s}^{\mathrm{PPO}}\approx 7$ )/LiTFSI, and poly­(epichlorohydrin) (PECH, $\varepsilon_{s}^{\mathrm{PECH}}\approx 15$ )/LiTFSI. While their molar conductivity follows the Walden rule, these polyether electrolytes exhibit intriguing features in glass transition and viscoelastic properties. In particular, BDS and rheology show clear slowing down of the structural relaxation time, *τ_α_*, along with a large elevation in glass transition temperature, *T*_g_, with salt concentration. DSC results, meanwhile, demonstrate a strong broadening of the *T*_g_ step or signs of phase separation. Interestingly, the elevation in *T*_g_ depends linearly on the salt concentration with a slope not correlating with their static dielectric constants. Furthermore, linear rheology shows an apparent “disentanglement” of these polymer electrolytes with salt concentration, while the separation between *τ_α_* and the terminal relaxation remain almost constant. We explain these results through two types of salt-induced structures correlating with their different ion solvation structures, which contribute differently to the glass transition and the linear viscoelastic properties: (i) the intrachain polymer-ion complex that shortens the effective chain length and (ii) the interchain polymer-ion complex that bridges different chains. These results point to a crucial role of the ion solvation structure in the local chain packing, which in turn, influences the polymer dynamics, glass transition, and ionic conductivity.

## Graphical abstract

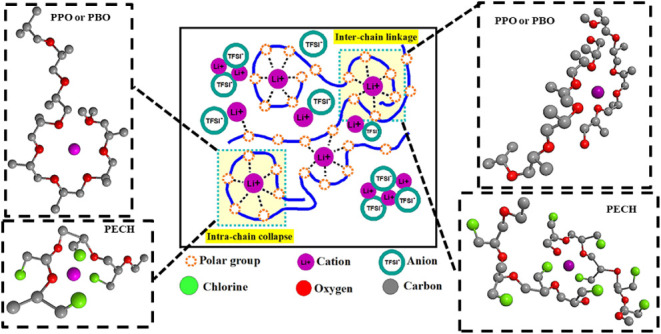

## 1 Introduction

Solid polymer electrolytes (SPEs) prepared through mixing polymers and salts have many advantages over small-molecule liquid electrolytes. For instance, SPEs are typically nonflammable, flexible, and have high-temperature stability.^1–4^ They are also compatible with lithium metal and thus can be used both for lithium-ion and lithium-metal batteries.^5–8^ Despite these widely acknowledged advantages of SPEs, one main challenge of SPEs is their relatively low ionic conductivity (10^–5^–10^–4^ S/cm) at ambient conditions, which is ∼10–10^2^ times smaller than the requirements for large-scale battery applications.^6,9,10^ From this perspective, strategies to design new SPEs with advanced performance are desired, which require a clear understanding of the influence of salt on polymer dynamics, glass transition, and ion transport.

Extensive efforts have been conducted in the past to understand the structure, dynamics, and ion transport of SPEs.^11–13^ Early and recent molecular dynamics simulations have suggested a combination of multiple pathways for ion transport in poly­(ethylene oxide) (PEO), including the Li^+^ subdiffusion along the PEO backbone, the movement of Li^+^ along with the PEO segment, and the Li^+^ hopping from one chain to its neighboring chains.^14–17^ A strong coupling has been observed between the ion diffusion and the segmental dynamics,^11,18–20^ which inspires various strategies to boost Li^+^ conductivity. On one hand, salt has to dissolve in the polymer matrix to produce free ions for ionic conductivity. Enhancing the free-ion concentration or lowering the glass transition has been actively explored to promote ionic conductivity.^9,21,22^ Along this line, the effect of dielectric constants of polymer matrices has been extensively discussed in bulk solid polymer electrolytes.^23–30^ Adding plasticizer or solvent to form gel polymer electrolytes has also been proposed to promote ion solvation or the segmental relaxation rates.^31,32^ On the other hand, strong polymer-ion interactions can lead to large elevations in *T*_g_, slowing down in segmental dynamics, and reduction in ionic conductivity. Optimum polymer-ion interactions have been suggested to balance the ion solvation and dynamics slowing down.^19,25–28,33^ Other methods, such as developing single-ion conductors,^34^ polymer blend electrolytes,^29,35,36^ composite polymer electrolytes,^37–39^ or imposing nanoconfinement,^40,41^ have also been pursued due to their suppression of ion–ion correlation (in the case of single-ion conductors), the creation of an additional pathway for Li^+^ diffusion (in composite polymer electrolytes), or a speeding up the segmental dynamics (through imposing nanoconfinement). Furthermore, SPEs that can decouple ion conductivity from structural relaxation have also shown promising room-temperature DC conductivity.^18,42^ Along with these important progresses, studies have revealed an intriguing role of polymer-ion interaction on ion clustering or macrophase separation.^43–46^ In particular, the relationship remains far from being understood between the polymer static dielectric constant, ion cluster formation, and their relationship with ionic conductivity.

In this work, we investigate the ion solvation structures, polymer dynamics, glass transition, and ionic conductivity of polyether electrolytes with different polarities, including poly­(butylene oxide) (PBO), poly­(propylene oxide) (PPO), and poly­(epichlorohydrin) (PECH). These polyethers have the same backbone structure as poly­(ethylene oxide) (PEO) and do not crystallize. They have different pendant groups (Figure 1), leading to their different average static dielectric constants of $\varepsilon_{s}^{\mathrm{PBO}}\approx 4.0$ for PBO, $\varepsilon_{s}^{\mathrm{PPO}}\approx 7.0$ for PPO, and $\varepsilon_{s}^{\mathrm{PECH}}\approx 15$ for PECH. This covers a range of polymer polarities weaker, comparable, and higher than that of PEO with $\varepsilon_{s}^{\mathrm{PEO}}\approx 8.0-9.0$ .^27^ Since this work focuses primarily on the influence of polymer polarity, we fix the salt as LiTFSI and vary the salt concentrations at [O]/[Li] (molar ratios) of 50:1, 15:1, and 5:1.

**Figure 1:**
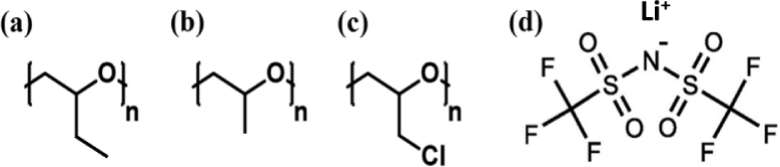
Molecular structures of the repeat units of (a) poly­(butylene oxide) (PBO), (b) poly­(propylene oxide) (PPO), (c) poly­(epichlorohydrin) (PECH), and (d) lithium bis­(trifluoromethanesulfonyl)­imide (LiTFSI).

X-ray scattering and Fourier transform infrared spectroscopy (FT-IR) found similar ion solvation structures of PPO/LiTFSI and PBO/LiTFSI to that of PEO/LiTFSI. In contrast, PECH/LiTFSI electrolytes exhibit different ion solvation structures from that of PEO/LiTFSI due to the presence of the chloride group. Broadband dielectric spectroscopy (BDS) and linear rheology measurements show a large influence of the salt on the segmental relaxation time, *τ_α_*, including a slowing down in *τ_α_* upon salt addition and an increment in the temperature dependence of *τ_α_*. At the same time, the DC conductivity of polymer electrolytes, *σ*_DC_, reduces with salt concentration from [O]:[Li] = 50:1 to 5:1. The Walden rule holds for all three types of electrolytes: a strong coupling between the molar conductivity, Λ, and *τ_α_*. Extrapolating to *τ_α_* = 100 s in BDS and rheology measurements gives dynamic glass transition temperatures, $T_{g}^{\mathrm{BDS}}$ or $T_{g}^{\mathrm{Rhg}}$ , which agree well with the differential scanning calorimetry (DSC) results, $T_{g}^{\mathrm{DSC}}$ . The superscripts BDS, Rhg, and DSC represent results from BDS, rheology, and DSC, respectively. At the same time, DSC measurements show clear broadening at high temperatures or a separated step in the reversible specific heat capacity, $C_{p}^{\mathrm{rev}}$ , for glass transition, highlighting an increment in dynamic heterogeneity or the presence of phase separation in the polymer electrolytes. Detailed analyses of the DSC show an interesting linear dependence of the largest shift in *T*_g_ and the salt concentration that has no direct correlation with the static dielectric constant of the polyether. In addition, linear rheology reveals the interesting influence of salt on chain dynamics and entanglement. In particular, the addition of LiTFSI enlarges *τ_e_*/*τ_α_* and impacts little *τ_f_*/*τ_α_* of the polymer electrolytes, where *τ_e_* and *τ_f_* are the characteristic entanglement relaxation time and the characteristic terminal relaxation time, respectively. As a result, the well-resolved rubbery plateau of the neat polymer shrinks progressively with salt concentration and leads to a complete elimination of the rubbery plateau at high salt loadings at [O]:[Li] = 5:1. These results signify an interesting synergy of the static dielectric constant and the salt concentration on the ion solvation structures, glass transition, entanglement dynamics, and ionic conductivity of polymer electrolytes. We explain them through two types of salt-induced structures: the salt-induced intrachain conformational changes and the ion-mediated interchain linkages that impact the glassy dynamics and the entanglement differently. Moreover, the formation of the intrachain conformation change and interchain linkage depends on the chemical structures and the location of the polar sites of the monomer, offering new insights for the future design of high-performance SPEs.

## 2 Materials and Methods

### 2.1 Materials

Poly­(butylene oxide) (PBO), poly­(propylene oxide) (PPO), and polyepichlorohydrin (PECH) with molecular weight of $M_{n}^{\mathrm{PBO}}=23.5\;\mathrm{kg}/\mathrm{mol}$ and polydispersity *Đ* = 1.16, $M_{n}^{\mathrm{PPO}}=19.8\;\mathrm{kg}/\mathrm{mol}$ and *Đ* = 1.04, and $M_{n}^{\mathrm{PECH}}=30.7\;\mathrm{kg}/\mathrm{mol}$ and *Đ* = 1.17 have been synthesized and characterized in a previous publication.^47^ Figure 1 gives a sketch of the repeat units of PBO, PPO, and PECH, and the chemical structure of Lithium bis­(trifluoromethanesulfonyl)­imide (LiTFSI). The three polymers have identical backbone structures but different pendant groups: ethyl, methyl, and chloromethyl groups. These different pendant groups lead to different static dielectric constants, $\varepsilon_{s}^{\mathrm{PBO}}\approx 4$ for neat PBO, $\varepsilon_{s}^{\mathrm{PPO}}\approx 7$ for neat PPO, and $\varepsilon_{s}^{\mathrm{PECH}}\approx 15$ for neat PECH, which cover a wide range of static dielectric constants that are smaller than, comparable to, and higher than the poly­(ethylene oxide) (PEO) with $\varepsilon_{s}^{\mathrm{PEO}}\approx 8-9$ .

The polymer electrolytes were prepared by mixing the neat polymer and the LiTFSI at the desired composition in tetrahydrofuran (THF) at room temperature with magnetic bar stirring for at least 2 h. After mixing, the solvent was evaporated under a fume hood, and the resulting polymer electrolytes were further dried in a vacuum oven (∼10^–3^ Pa) at *T* = 333 K for a minimum of 72 h, followed by an additional drying step at *T* = 360 K for at least another 24 h at the same vacuum level (∼10^–3^ Pa). The samples were then stored in a vacuum jar filled with desiccants (Drierite 21005 indicating desiccant, 4 Mesh, Cole-Parmer). Prior to each measurement, the samples were subjected to a final drying step in the vacuum oven (∼10^–3^ Pa) at *T* = 333 K.

### 2.2 X-ray Scattering

Small-angle X-ray scattering (SAXS) and wide-angle X-ray scattering (WAXS) were performed to characterize the structure of the neat polymer and the polymer electrolytes. All the measurements were conducted at Beamline 12-ID-B at the Advanced Photon Source of Argonne National Laboratory with a 2D Pilatus 2M detector. The wavevector *Q* values of the detector pixels were calibrated using silver behenate before the measurements. The sample-to-detector distance was 2.0 m, and the X-ray energy was 13.3 keV with a wavelength λ = 0.9347 Å. In all measurements, the sample was sealed in a Tzero Hermetic pan with a lid (TA Instruments). An empty Tzero Hermetic pan was measured as a background, and its signal was subtracted from the scattering intensity to obtain the polymer signal. For each polymer electrolyte, we sampled 10 different locations of each sample to make sure the results were reproducible. The 2D isotropic scattering images were converted to a 1D X-ray scattering intensity curve *I*(*Q*) vs *Q* through azimuthal averaging after solid angle correction and then normalized with the intensity of the transmitted X-ray beam flux, using the beamline software.

### 2.3 Fourier Transform Infrared Spectroscopy (FT-IR)

Fourier-transform infrared spectroscopy (FT-IR) measurements were performed at T = 293 K by using a ThermoFisher Nicolet iS50 spectrometer with a Harrick Praying Mantis diffuse reflectance IR Fourier transform spectroscopy (DRIFTS) accessory. Spectra were recorded from 4000 to 400 cm^–1^, averaging 100 scans at a resolution of 4 cm^–1^. IR-grade KBr powder (ThermoFisher) was used as the background, and all samples were mounted directly on top of the KBr base during the measurement to avoid any other signal interfering with the sample signal.

### 2.4 Temperature-Modulated Differential Scanning Calorimetry (TMDSC)

Temperature-modulated differential scanning calorimetry (TMDSC) was carried out using a Discovery Q50 (TA Instruments) from 353 to 183 K at a cooling rate of 2 K/min and a modulation amplitude of ±1 K every 60 s. For each measurement, ∼6 mg of sample was put in a Tzero aluminum pan that was capped with a Tzero aluminum lid. An isothermal step at 353 K for 5 min was applied before the cooling and modulation. The glass transition temperatures, *T*_g_, were identified as the inflection point of the glass transition step upon cooling, which corresponds to the peak of the $dC_{p}^{\mathrm{rev}}/dT$ curve with $C_{p}^{\mathrm{rev}}$ being the reversible specific heat capacity.

### 2.5 Broadband Dielectric Spectroscopy (BDS)

Broadband dielectric spectroscopy (BDS) has been applied to characterize the polymer dynamics and the DC conductivity of polymer electrolytes. The dielectric responses were measured in a frequency range of 10^–2^–10^7^ Hz on a Novocontrol Concept 40 system, which includes an Alpha-A impedance analyzer, a ZGS active sample cell interface, and a Quatro Cryosystem temperature control system. The temperature accuracy is ±0.1 K. In all measurements, the polyether electrolytes were sandwiched between two gold-plated electrodes with a diameter of 20 mm, along with a hollow Teflon ring spacer with an inner diameter of 16 mm, an outer diameter of 25 mm, and a thickness of L = 0.14 mm. The root-mean-square of the applied alternating current (AC) voltage was 0.1 V. For each sample, the measurements covered a temperature range of *T*_g_+ 100 K to *T*_g_ – 10 K at an interval of 5 K upon cooling, and a temperature range from *T*_g_ – 10 K to *T*_g_ + 100 K at an interval of 10 K upon heating to check the reproducibility of the measurements. All electrolytes were dried under high vacuum (∼10^–3^ Pa) at 313 K for at least 24 h to remove the moisture. Before measurements at each temperature, a thermal annealing of 20 min was applied to ensure the thermal equilibrium of the sample at the testing temperature.

### 2.6 Rheology

Small amplitude oscillatory shear (SAOS) measurements were performed on an Anton Paar MCR 302 instrument, along with a CTD 600 environmental oven and an EVU 20 liquid nitrogen evaporation unit. A pair of parallel aluminum plates with a diameter of 4 mm and a strain amplitude of 0.1% was used to access the glassy region with a modulus of ∼10^9^ Pa close to *T*_g_. For dynamic shear modulus lower than 10^6^ Pa, a pair of aluminum plates with a diameter of 8 mm and a strain amplitude of 1–5% was used. For each measurement, the samples were dried in a vacuum oven (∼10^–3^ Pa) for at least 24 h before measurements. The sample loading onto the rheometer was accomplished at high temperatures, i.e, *T*_g_ + 100 K, and was gradually cooled down to temperatures close to *T*_g_ for testing. No universal temperature range was considered for the rheological measurements due to the different *T*_g_s of these polymer electrolytes. The measurements were terminated when terminal modes were well visible. A thermal annealing of 20 min was applied before each measurement to ensure thermal equilibrium. The gap was fixed at ∼1 mm, and the dynamic frequency range was 10^2^ to 10^–1^ rad/s. Linear viscoelastic master curves were then constructed through the time–temperature superposition principle.

## 3 Results and Discussions

### 3.1 X-ray Scattering and Fourier Transform Infrared Spectroscopy

X-ray scattering measurements were performed to characterize the ion solvation structures of polymer electrolytes. Figure 2 presents the shifted X-ray scattering intensity, *I*(*Q*), at wavevector *Q* = 0.1–2.6 Å^–1^ for the neat polymers and the electrolytes of PECH/LiTFSI (Figure 2a), PPO/LiTFSI (Figure 2b), and PBO/LiTFSI (Figure 2c). Note that the X-ray measurements cover a much wider *Q* range down to 0.005 Å^–1^, and no structures have been observed at *Q* < 0.1 Å^–1^. Hence, we only present the data at *Q* = 0.1–2.6 Å^–1^. Two scattering peaks are observed: (i) a main peak at *Q*_1_ ≈ 1.4–1.5 Å^–1^; and (ii) a shoulder peak at *Q*_2_ ≈ 0.75–1.0 Å^–1^. The main scattering peaks at *Q*_1_ ≈ 1.4–1.5 Å^*–*1^ have been observed in neat amorphous PEO as well, which has been attributed to the correlation between the chain backbone oxygen.^48,49^ Interestingly, the neat PEO does not show a shoulder peak at *Q* ≈ 0.75–1.0 Å^–1^ like the PPO, PBO, and PECH, implying the side group’s influence to the scattering.^49^

**Figure 2:**
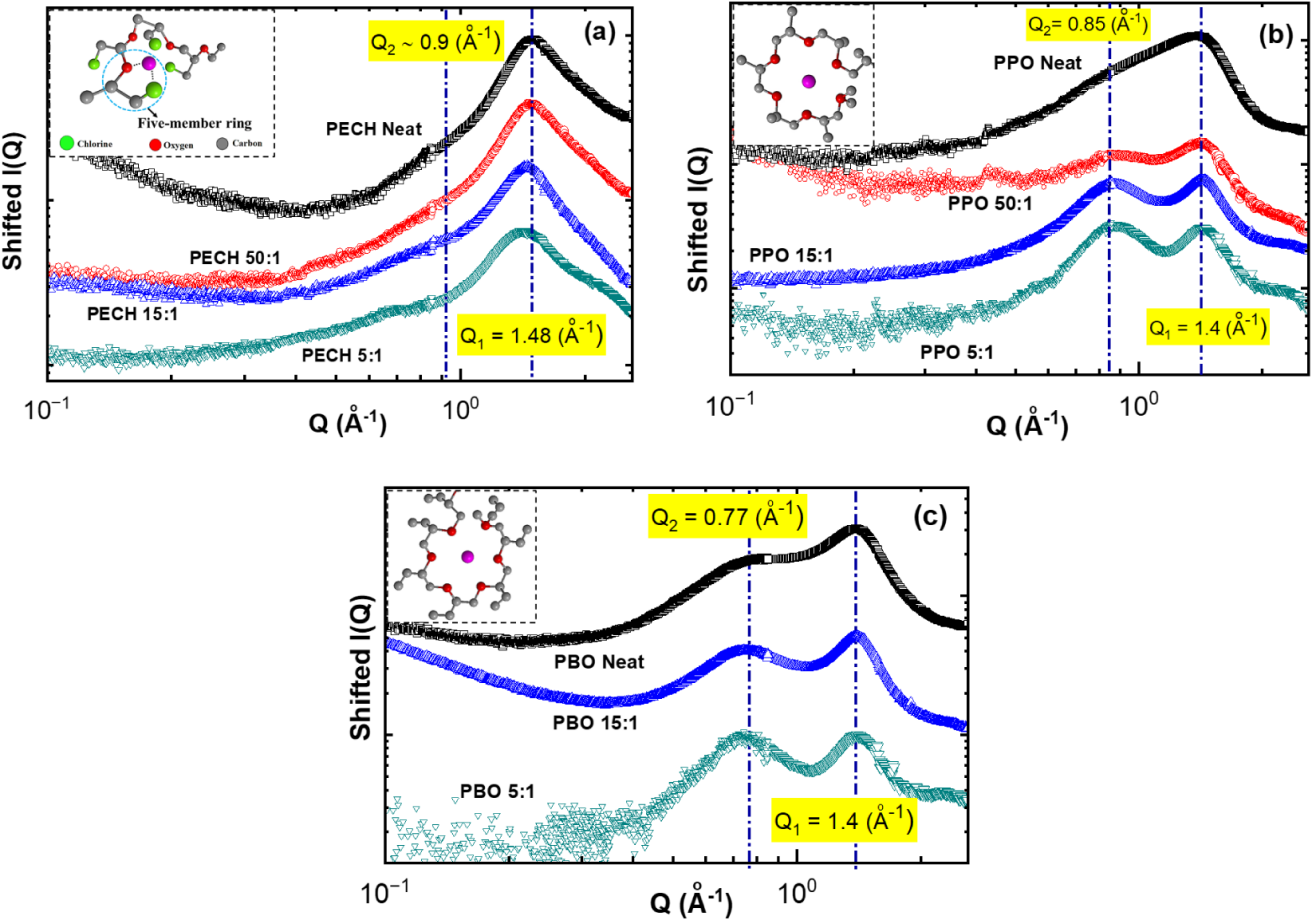
X-ray scattering of (a) neat PECH and PECH/LiTFSI, (b) neat PPO and PPO/LiTFSI, and (c) neat PBO and PBO/LiTFSI at room temperature (293 K). The inset shows (a) a sketch of a five-membered-ring ion solvation structure that might exist in PECH/LiTFSI, (b) a sketch of the crown-ether ion solvation structure of PPO/LiTFSI, and (c) a sketch of the crown-ether ion solvation structure of PBO/LiTFSI. The atom color scheme of the inset cartoon follows that of Figure 2a.

Adding LiTFSI leads to several changes in the X-ray scattering spectra. First, the scattering peak at *Q*_1_ ≈ 1.4–1.5 Å^–1^ becomes slightly narrower for PPO/LiTFSI and PBO/LiTFSI, which has been observed in PEO/LiTFSI and has been attributed to the formation of crown-ether structures (inset of Figure 2b and c) through backbone oxygen coordinating with Li^+^.^49^ From this perspective, PPO/LiTFSI and PBO/LiTFSI exhibit similar ion solvation structures as PEO/LiTFSI. This is expected since PPO and PBO do not have any polar side groups. Similar to PEO/LiTFSI, the intensity of the shoulder peak at *Q* ≈ 0.75–1.0 Å^*–*1^ increases with salt addition for PPO/LiTFSI and PBO/LiTFSI, which becomes pronounced at [O]:[Li] = 15:1 or [O]:[Li] = 5:1. The higher the salt concentration, the stronger the peak intensity. Recent studies suggest the peak at *Q* ≈ 0.75–1.0 Å^*–*1^ corresponds to the anion–anion correlation in PEO/LiTFSI. These features suggest that similar ion solvation structures between PPO/LiTFSI, PBO/LiTFSI, and PEO/LiTFSI.^48,49^

On the other hand, the inclusion of LiTFSI to PECH does not lead to the narrowing of the peak *Q* ≈ 1.4–1.5 Å^–1^ or an increase in scattering intensity at *Q* ≈ 0.75–1.0 Å^*–*1^, indicating their different ion solvation structures from PPO/LiTFSI and PBO/LiTFSI. These observations suggest a strong impact of chloride on the ion solvation structures. For instance, the chloride of PECH can combine with the backbone oxygen to coordinate with Li^+^, forming a five-membered ring structure (inset of Figure 2a). The possibility of this five-membered ring structure formation provides a different ion solvation structure of PECH/LiTFSI, in addition to the crown-ether structures from the Li^+^···O interaction of the backbone oxygen. We notice that a similar weak influence of salt on the shoulder peak at *Q* lower than the main scattering peak has recently been observed in poly­(pentyl malonate) (PPM)/LiTFSI, which has been attributed to different ion solvation structures from the classical crown-ether structures.^48^ A similar scenario might happen in PECH/LiTFSI, and future computer simulations should help resolve the possible ion solvation structures of PECH/LiTFSI.

To characterize further the ion solvation structures, we have conducted Fourier transform infrared spectroscopy (FT-IR) measurements, focusing on the C–O–C stretching that reflects the influence of ion solvation.^50,51^ As shown in Figure 3a, LiTFSI leads to a slight blue shift of the C–O–C stretching from the neat PECH at 1153 cm^–1^ to 1155 cm^–1^ of PECH/LiTFSI at [O]:[Li] = 5:1. In contrast, the C–O–C stretching of neat PPO exhibits a large red shift from 1172 cm^–1^ of neat PPO to 1150 cm^–1^ of PPO/LiTFSI at [O]:[Li] = 5:1. Similar red shifts in the C–O–C stretching of PBO have been observed, from 1159 cm^–1^ of the neat PBO to 1150 cm^–1^ of PBO/LiTFSI at [O]:[Li] = 5:1. The red shifts in the C–O–C stretching upon salt addition have been widely observed in polyether electrolytes, such as PEO/LiTFSI, and have been associated with crown-ether structures, which involve strong conformational changes of the polyether chain backbone.^50^ The large red shifts in the C–O–C stretching of PPO/LiTFSI and PBO/LiTFSI are consistent with the X-ray scattering results on the leading ion solvation structures of the crown-ether structures. On the other hand, there are negligible changes in the C–O–C stretching (or a slight blue shift) of PECH/LiTFSI with salt concentrations, suggesting the presence of different types of ion solvation structures. This observation is consistent with the X-ray scattering measurements of the PECH/LiTFSI electrolytes and supports the conclusion of a different ion solvation structure for PECH/LiTFSI compared to PPO/LiTFSI and PBO/LiTFSI. Furthermore, the lack of red shifts in the C–O–C stretching of PECH/LiTFSI implies a small influence of Li^+^ ion solvation on the local chain backbone conformation, which aligns with the proposed five-member ring ion solvation structure (Inset of Figure 2a) of PECH/LiTFSI. Nevertheless, the combined X-ray scattering and FT-IR measurements point out (i) that the ion solvation structures of PPO/LiTFSI and PBO/LiTFSI are dominated by crown-ether types of ion solvation structures, and (ii) that PECH/LiTFSI electrolytes exhibit a different ion solvation structure from the crown-ether structures, highlighting an interesting role of the chloride group in ion solvation.

**Figure 3:**
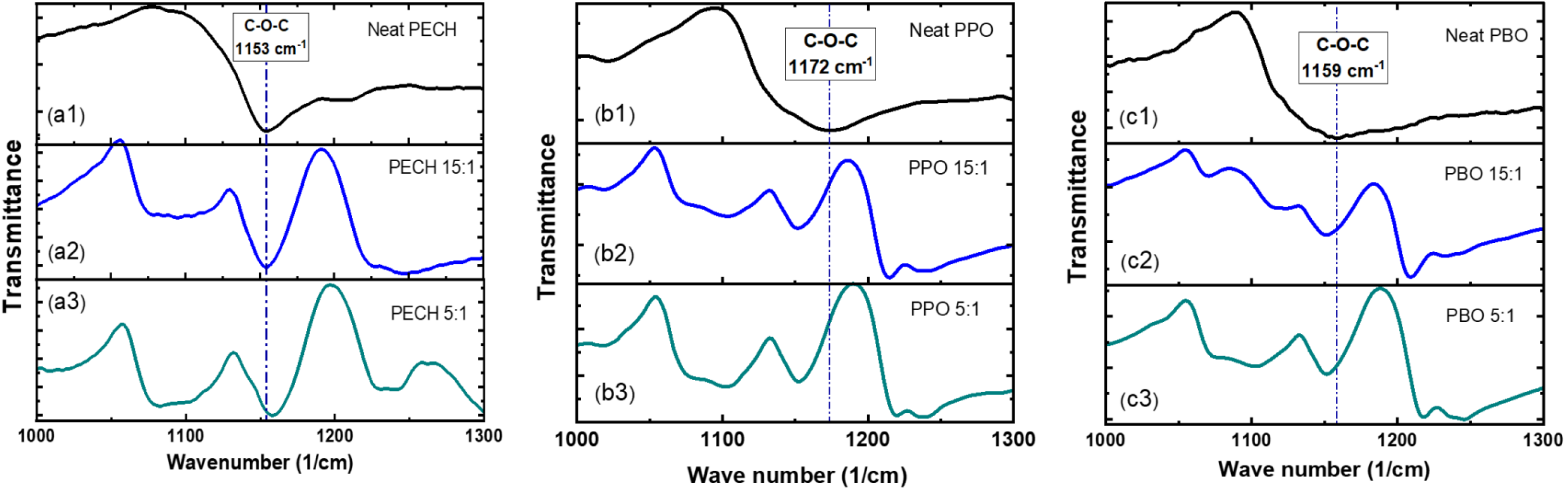
Fourier transform infrared spectroscopy (FT-IR) spectra of the neat polymers and the polymer electrolytes at 1000–1300 cm^–1^, where the C–O–C stretching is observed. (a) Neat PECH and PECH/LiTFSI electrolytes, (b) neat PPO and PPO/LiTFSI, and (c) neat PBO and PBO/LiTFSI. The measurements were conducted at room temperature (293 K).

### 3.2 Differential Scanning Calorimetry

Figure 4a–c provides the reversible specific heat capacity, $C_{p}^{\mathrm{rev}}$ , of PECH/LiTFSI, PPO/LiTFSI, and PBO/LiTFSI, respectively, at [O]:[Li] = 50:1, 15:1, and 5:1. The $C_{p}^{\mathrm{rev}}$ of neat PECH, neat PPO, and neat PBO are presented for comparison. The derivatives of $C_{p}^{\mathrm{rev}}$ with temperature, $dC_{p}^{\mathrm{rev}}/dT$ , for the corresponding electrolytes and the neat samples are presented in Figure 4d–f. Table 1 summarizes the onset (*T*_g–Onset_, defined as the onset temperature of the $dC_{p}^{\mathrm{rev}}/dT$ peak) and the end (*T*_g–End_, defined as the end of the $dC_{p}^{\mathrm{rev}}/dT$ peak) of the glass transition step, as well as the characteristic *T*_g_ defined through the $dC_{p}^{\mathrm{rev}}/dT$ peak of the polymer electrolytes and the neat polymers. Inclusion of LiTFSI leads to several interesting changes in the glass transition. One or two *T*_g_s can be observed in these electrolytes. For PBO/LiTFSI, two *T_g_* s have been observed for [O]:[Li] = 15:1, and one *T*_g_ was found for [O]:[Li] = 5:1. One *T*_g_ was observed for PPO/LiTFSI and PECH/LiTFSI with [O]:[Li] = 50:1 and 15:1, and two *T*_g_s for [O]:[Li] = 5:1. The presence of two *T*_g_s suggests macrophase separation. Interestingly, the [O]:[Li] ratio where PBO/LiTFSI undergoes macrophase separation is quite different from that of PPO/LiTFSI and PECH/LiTFSI, implying an influence of the polyether type on the phase behaviors. For polymer electrolytes with one *T*_g_, a broadening in *T*_g_ step (compared with the neat parent polymer) has often been observed (see Figure 4d–f), suggesting an increment in the dynamic heterogeneity of these polymer electrolytes. Another interesting feature signifying the increment in heterogeneity is the broadening of the $dC_{p}^{\mathrm{rev}}/dT$ at the high-temperature side of the *T*_g_ step (an elevation in *T*_g_). Despite the clear macrophase separation and the increment in dynamic heterogeneity of the polymer electrolytes from the DSC measurements, the X-ray scattering spectra do not show structures beyond the two amorphous halo peaks shown in Figure 2. This might be due to a lack of electron density contrast between different phases of the polymer electrolytes with macrophase separation.

**Figure 4:**
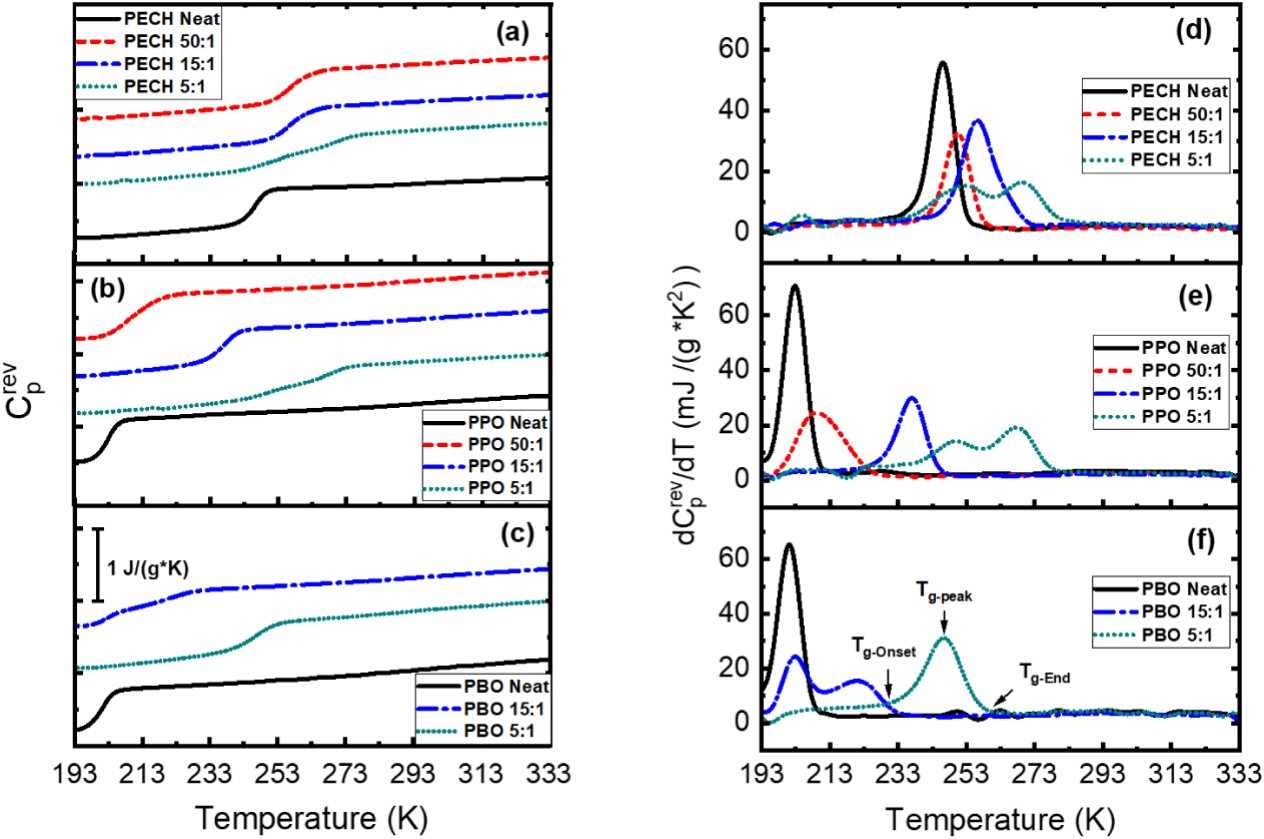
Reversible specific heat capacity, $C_{p}^{\mathrm{rev}}$ , of (a) PECH/LiTFSI, (b) PPO/LiTFSI, and (c) PBO/LiTFSI, and their first derivative with respect to temperature, $dC_{p}^{\mathrm{rev}}/dT$ , of (d) PECH/LiTFSI, (e) PPO/LiTFSI, and (f) PBO/LiTFSI. The corresponding data of neat PECH, neat PPO, and neat PBO are presented as solid black lines for comparison.

**Table 1:** Glass Transition Temperature of the Polyether Electrolytes

Polymer	O:Li	$$T_{g-\mathrm{Onset}}\left(K\right)$$	$$T_{g-\mathrm{End}}\left(K\right)$$	$T_{g-B.W}\left(K\right)$ ^a^	$T_{g}^{\mathrm{DSC}}\left(K\right)$ ^b^	$T_{g}^{\mathrm{BDS}}\left(K\right)$ ^c^	$T_{g}^{\mathrm{Rhg}}\left(K\right)$ ^d^
**PECH**	Neat	233	257	24	246	242	243
	50:1	239	259	20	250	251	247
	15:1	243	272	29	257	256	258
	5:1	236	281	45	251, 270	270	271
**PPO**	Neat	193	211	17.5	204	202	—–
	50:1	196	226	30	211	216	—–
	15:1	222	247	25	237	235	235.5
	5:1	237	279	42	250, 268	263	265
**PBO**	Neat	193	210	17	202	200	—–
	15:1	196	234	38	204, 221	219	210
	5:1	228	261	33	247	254	254

a $T_{g-B.W}=T_{g-\mathrm{End}}-T_{g-\mathrm{Onset}}$ .

b *T*_g_ measured from DSC measurement.

c *T*_g_ estimated from BDS measurement (±4 K).

d *T*_g_ estimated from Rheology measurement (±4 K).

The macrophase separation of the polyether/LiTFSI electrolytes has been observed previously.^43^ Although an explicit description of the phase behavior of polymer electrolytes is not available,^52^ important insights can be obtained from comparing the key length scales associated with the electrolyte systems.^53^ Assuming 100% ion solvation, one can estimate the average ion spacing, $r_{\mathrm{ion}}={\left(\frac{3}{4\pi n_{p}}\right)}^{1/3}$ , where $n_{p}=\frac{\rho_{LiTFSI}N_{A}}{M_{LiTFSI}}$ is the number density of ion pairs with *M*_LiTFSI_ and *ρ*_LiTFSI_ being the molecular weight and the mass density of LiTFSI.^54^ Thus, *r*_ion_ exhibits weak temperature dependence. The Bjerrum length, $l_{B}=\frac{e^{2}}{4\pi \varepsilon_{e}\varepsilon_{0}k_{B}T}$ , and the Keesom length, $r_{K}={[\frac{p^{2}}{2\sqrt{6}\pi \varepsilon_{e}\varepsilon_{0}k_{B}T}]}^{1/3}$ , can be estimated from the *static dielectric constant* of the electrolytes, *ε_e_*, where *e* is the charge unit, *ε*_0_ the vacuum permittivity, *k*_B_ the Boltzmann constant, *T* the absolute temperature,^53,55^ and *p* ≈ 10 *D* is the dipole moment of the ion pair.^56,57^ The Bjerrum length and Keesom length are estimated by comparing the thermal energy *k*_B_ *T* with the electrostatic potential energy between two ions and between two ion pairs, respectively. In other words, the Bjerrum length represents the shortest distance between neighboring ions to keep them solvated, and the Keesom length represents the shortest distance below which ion pairs remain in their aggregated states. Therefore, the Bjerrum length and Keesom length depend on the temperature. In general, when *r*_ion_ < *r_K_*, ions remain mostly nondissociated, i.e., the so-called ionomer region. When *r*_ion_ > *l_B_*, ions are in the dispersed state, and the system is in a polyelectrolyte region. At the intermediate length scales, *r_K_* < *r*_ion_ < *l_B_*, the ionomer phase and the polyelectrolyte phase coexist with structural heterogeneity in the polymer electrolytes. We would like to emphasize that the comparisons of *r_K_*, *r*_ion_, and *l_B_* only provide a qualitative description of the possible structures of the polymer electrolytes.

Figure 5 gives the representative estimate of *r*_ion_ and their comparison with *r_K_* and *l_B_* for PECH/LiTFSI at [O]:[Li] = 15:1. Figure S1 represents results for the PECH/LiTFSI electrolytes at [O]:[Li] = 5:1 and 50:1, as well as the results for PBO/LiTFSI and PPO/LiTFSI. *r_K_* < *r*_ion_ < *l_B_* has been observed for all polymer electrolytes at all three salt concentrations, implying a high chance of the coexistence of the ionomer phase and the polyelectrolyte phase in these electrolytes. We attribute the coexistence of the ionomer phase and the polyelectrolyte phase to the strong broadening of the *T*_g_ or the two separated *T*_g_s of these polyether electrolytes.

**Figure 5:**
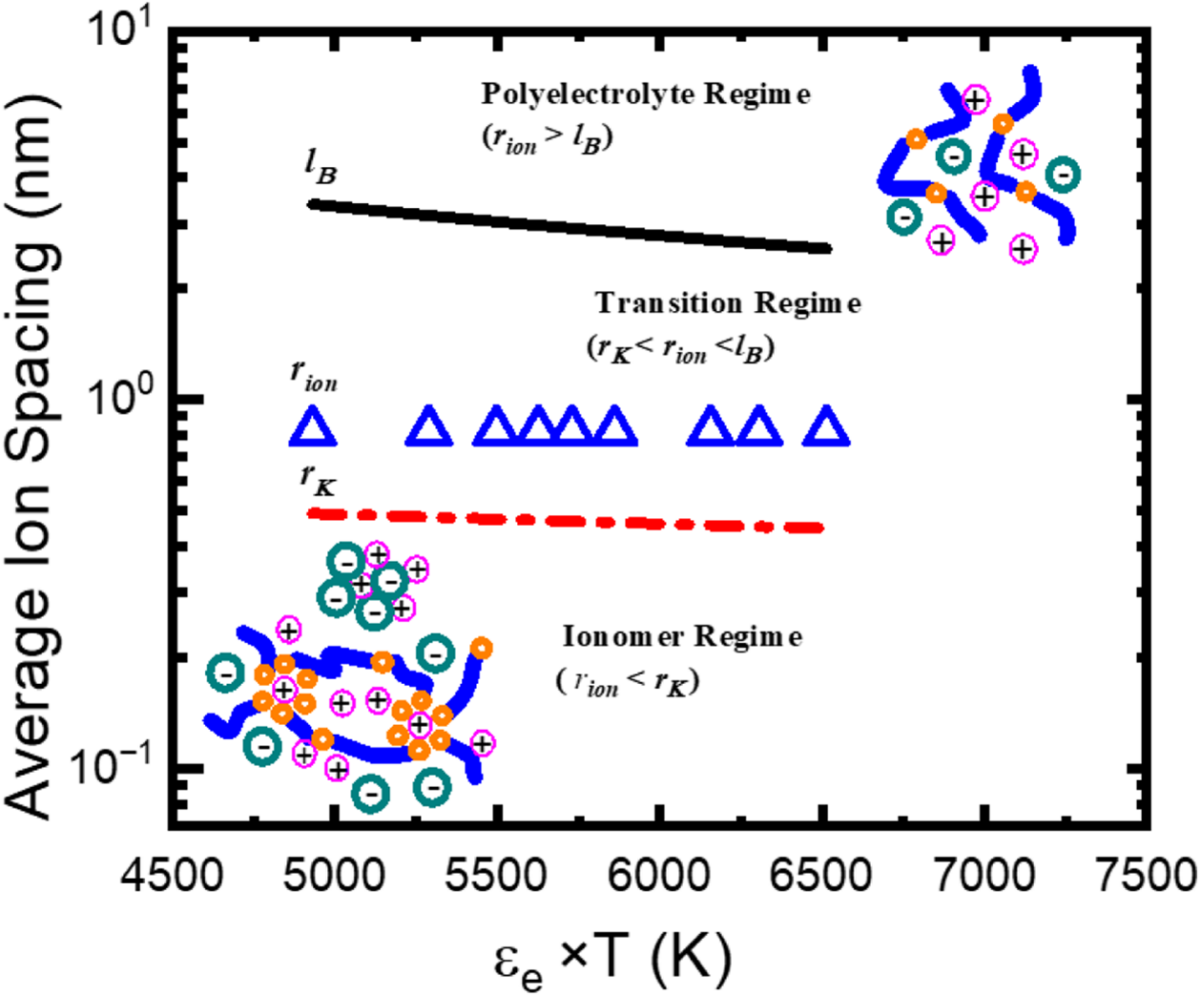
Bjerrum length, *l*_*B*_ (solid lines), Keesom length, *r*_*K*_ (dashed lines), and average ion spacing, *r*_ion_ (symbols) over ε_e_ × *T* of PECH/LiTFSI electrolyte at [O]:[Li] = 15:1.

We further analyze the shift in the glass transition temperature of the polymer electrolytes, Δ*T*_g_ *= T*_g_ *–* T_g,Neat_, where *T*_g,Neat_ represents the glass transition temperature of the neat polymer. In particular, *T*_g_ of PBO/LiTFSI increases from 202 K of the neat PBO to ∼246 K of PBO/LiTFSI at [O]:[Li] = 5:1, offering an Δ*T*_g_ ∼ 44 K increment. A similar increment in *T*_g_ has been observed for PPO/LiTFSI from ∼203 K of the neat PPO to ∼248 and 263 K at [O]:[Li] = 5:1. Compared with PBO and PPO, adding LiTFSI into PECH results in a much less change in *T*_g_, with Δ*T*_g_ ∼ 4–17 K from 246 K of the neat PECH to ∼250 and 263 K at [O]:[Li] = 5:1. Plotting the Δ*T*_g_ against the volume fraction of the salt (Figure 6), *ϕ*(%), gives several interesting observations: (i) A near-linear dependence between Δ*T*_g_ and *ϕ*(%) has been observed. The strong dependence of Δ*T*_g_ on salt concentration is reminiscent of the glass transition in cross-linked polymers or associative polymers.^58^ This might be understood through the long-lived (compared with the structural relaxation of the polymer) polymer-ion association that can temporarily cross-link the polymer. (ii) The slopes of the Δ*T*_g_ vs *ϕ*(%) change with the polyether types, implying a strong effect of the matrix polymer on the *T*_g_ of polymer electrolytes. Importantly, Δ*T*_g_ has the following order: PPO > PBO > PECH at a given salt loading, which does not agree with the order of their static dielectric constants. These results suggest that the static dielectric constant of the matrix polymer does not necessarily correlate with the glass transition of polymer electrolytes.

**Figure 6:**
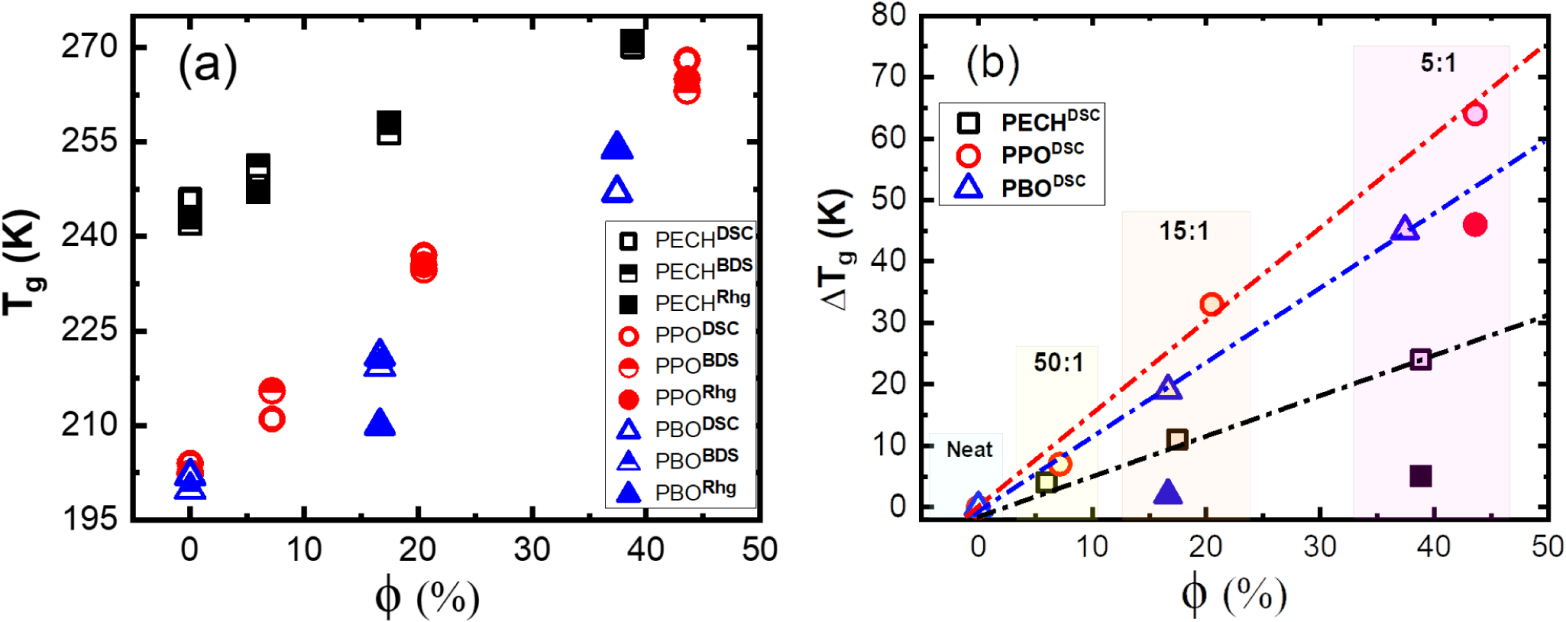
(a) The glass transition temperature *T*_g_ measured from DSC (open symbols with superscript of DSC), estimated from VFT fits to τ_α_ from BDS test (half-filled symbols with superscript of BDS), and estimated from VFT fits to τ_α_ from rheology (filled symbols with superscript of Rhg), and (b) the shift in glass transition, Δ*T*_g_ = *T*_g_–*T*_g,Neat_, (from DSC test) over the salt volume fraction ϕ(%) for all of the electrolyte systems. The filled symbols in (b) represent the low-temperature *T*_g_ for the phase-separated systems. A nearly linear response of Δ*T*_g_ over ϕ(%) is observed with slopes of ∼0.43 K/%, ∼1.47 K/%, and ∼0.78 K/% for PECH/LiTFSI, PPO/LiTFSI, and PBO/LiTFSI polymer electrolyte systems, respectively. The mass densities of LiTFSI, PECH, PPO, and PBO are 1.33 g/cm³, 1.36 g/cm³, 1.04 g/cm³, and 1.00 g/cm³, respectively.

### 3.3 Broadband Dielectric Spectroscopy (BDS)

#### 3.3.1 General Features

Figure 7a,b shows the representative dielectric storage permittivity, *ε*′(*ω*), loss permittivity, *ε*′′(*ω*), and the derivative spectra, $\varepsilon_{\mathrm{der}}^{’}\left(\omega \right)=-\frac{\pi }{2}\frac{\partial \varepsilon^{\prime }\left(\omega \right)}{\partial \ln\omega }$ , of neat PECH and PECH/LiTFSI at [O]:[Li] = 15:1 and *T* = 303 K. BDS spectra of other PECH/LiTFSI, PPO/LiTFSI, and PBO/LiTFSI samples are provided in Figure S2. The PECH, PPO, and PBO are all type-A polymers and should have normal modes in the dielectric measurements.^59^ However, the strong DC conductivity and the high dielectric amplitude of the Maxwell–Wagner–Sillars’ (MWS) interfacial polarization process shield the dielectric normal modes of the neat polymers and the polymer electrolytes.^59^ In particular, the LiTFSI leads to several modifications in the dielectric properties (Figure 7): (i) The segmental dynamics of polymer electrolytes slow down significantly compared with the neat polymer. (ii) The dielectric dispersion of the structural relaxation of polymer electrolytes is much more broadened than that of the neat matrix polymer. (iii) The polymer electrolytes have a significantly higher *static dielectric constant* than the neat polymer, *ε_e_* > *ε_s_*, where *ε_s_* is the static dielectric constant of the neat polymer. For the neat polymer and the polymer electrolytes, the static dielectric constants (*ε_e_* or *ε_s_*) are determined through the plateau of the dielectric storage permittivity at angular frequencies before the rise of the electrolyte polarization (EP) as shown by the short dashed line labeling in Figure 7a. This value is equal to the sum of the dielectric relaxation amplitude, Δε, of all molecular relaxation processes plus the dielectric constant of the material at infinitely high frequency, *ε*_∞_. (iv) *σ*_DC_ of polymer electrolytes are much higher than that of the neat polymer. (v) The polymer electrolytes exhibit a strong EP process, offering important characterizations of parameters controlling the DC conductivity. These features agree well with previous dielectric measurements of polyether electrolytes.^42,43,60^ Below, we provide detailed analyses of the dielectric properties.

**Figure 7:**
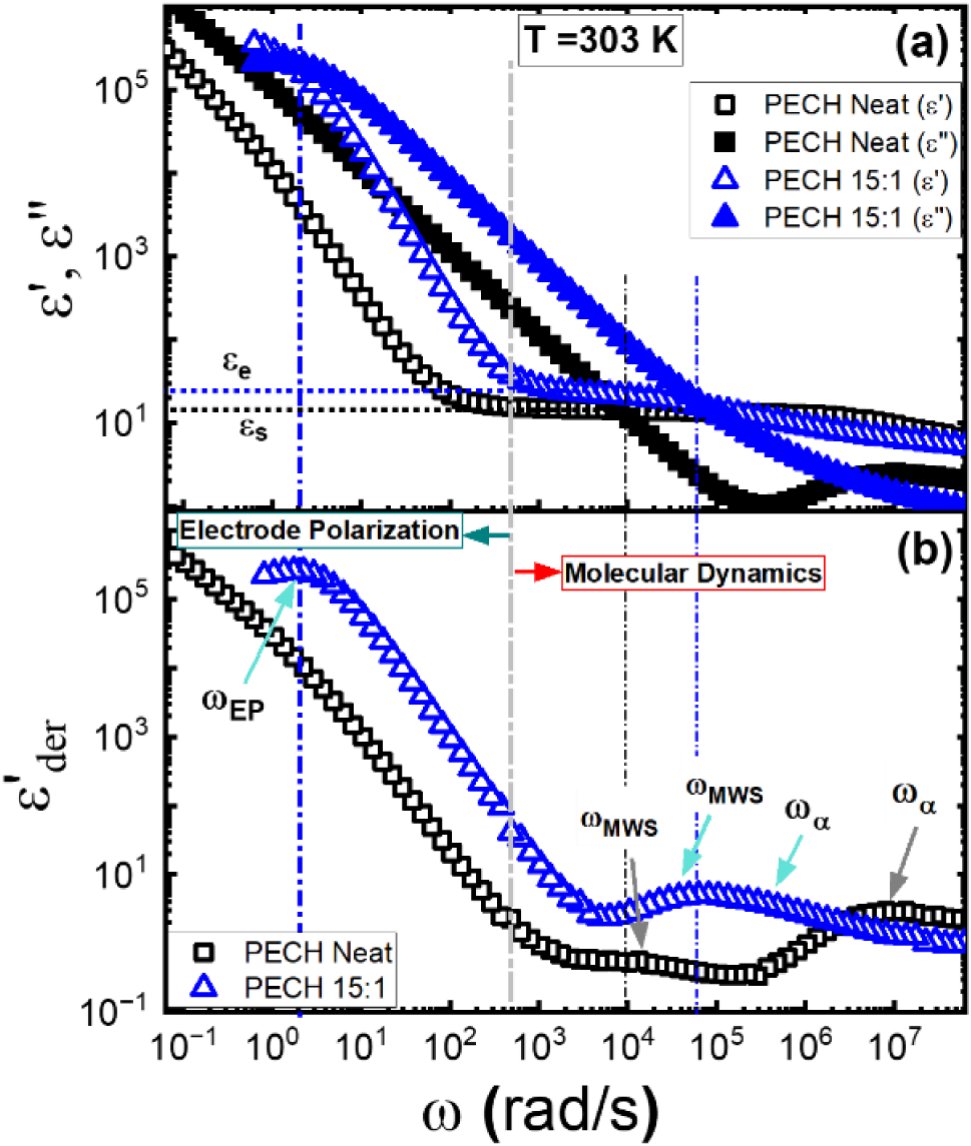
Dielectric spectrum, (a) ε′(ω), ε″(ω), and (b) $\varepsilon_{\mathrm{der}}^{\prime }\left(\omega \right)$ , of neat PECH and PECH/LiTFSI at 15:1 at 303 K. The segmental relaxation process ω_α_, the Maxwell–Wagner–Sillars (MWS) process ω_MWS_, the electrode polarization process ω_EP_, static dielectric constant of the electrolyte ε_e_, and the neat polymer ε_*s*_ are depicted. The electrode polarization and molecular dynamic regions are separated in (b).

#### 3.3.2 The Electrode Polarization

Electrolytes interacting with metal electrodes form an electric double layer (EDL) at the electrolyte/electrode interface. The dielectric properties of the EDL involve an active dielectric relaxation: electrode polarization (EP). In particular, the complex permittivity of EP $\varepsilon_{\mathrm{EP}}^{*}\left(\omega \right)$ can be described by a Debye process as expressed in Equation 1:^61,62^

1 $$\varepsilon_{\mathrm{EP}}^{*}\left(\omega \right)=\varepsilon_{e}+\frac{\Delta \varepsilon_{\mathrm{EP}}}{1+i\omega \tau_{\mathrm{EP}}}$$

where Δ*ε*_EP_ is the dielectric relaxation strength, and *τ*_EP_ is the characteristic relaxation time of the EP process. Typically, one fits the loss factor tan*δ*(*ω*) of the EP process derived from Equation 1,

2 $$\tan\delta \left(\omega \right)=\varepsilon^{\prime \prime }\left(\omega \right)/\varepsilon^{\prime }\left(\omega \right)≅\frac{\omega \tau_{\mathrm{EP}}}{1+\omega^{2}{\left(\tau_{\mathrm{EP}}/\sqrt{M}\right)}^{2}}$$

with *M*=(Δ*ε*_EP_ + *ε_e_*)/*ε_e_* and when $\Delta \varepsilon_{\mathrm{EP}}\omega \tau_{\mathrm{EP}}\gg \left(\sigma_{\mathrm{DC}}/\omega \varepsilon_{0}\right)\left(1+\omega^{2}\tau_{\mathrm{EP}}^{2}\right)$ . *ε*_0_ is the vacuum permittivity and *ω* is the angular frequency. Thus, the characteristic peak of tan *δ*(*ω*) shifts $\sqrt{M}$ times to higher frequencies than the EP process of the dielectric loss spectra $\varepsilon_{\mathrm{EP}}^{’’}\left(\omega \right)$ . The analysis provides *M*, *ε_e_*, Δ*ε*_EP_, and *τ*_EP_. As a result, one can obtain the characteristic relaxation time of the conductivity relaxation, *τ*_ion_= *τ*_EP_/*M*.

Figure 8a,b shows the representative EP processes and the loss factor, tan*δ*(*ω*), of PECH/LiTFSI at [O]:[Li] = 5:1 at *T* = 393 K, along with the fit (the solid lines) to Equations 1 and 2. Figure 8c–f presents similar analyses for PPO 15:1 and PBO 5:1 at *T* = 313 K, respectively. We assign *ω*_EP_ as the characteristic angular frequency of the EP peak and *ω*_max_ as the peak frequency of tan *δ*(*ω*). Clear shift in the *ω*_EP_ to a higher frequency, *ω*_max_, were observed in all polymer electrolytes. Figure 9a–c summarizes *M* (Figure 9a), *τ*_ion_ (Figure 9b), and *ε_e_* (Figure 9c) of these polymer electrolytes and their dependence on temperature, where Δε_EP_ is given in the inset of Figure 9a. $\frac{\omega_{\max}}{\omega_{\mathrm{EP}}}∼\sqrt{M}$ holds for all polymer electrolytes (inset of Figure 9b), which is consistent with the McDonald model.^61,62^ The *τ*_ion_ = *τ*_EP_/*M* follows the Vogel–Fulcher–Tammann (VFT) temperature dependence, which links to the DC conductivity via the Barton–Nakajima–Namikawa (BNN) relationship, $\sigma_{\mathrm{DC}}^{\mathrm{BNN}}=C\Delta \varepsilon_{\mathrm{EP}}\varepsilon_{0}/\tau_{\mathrm{ion}}$ , with *C* being a constant prefactor.^63,64^ As shown in Figure 9c, compared with the neat polymers (filled symbols), the polymer electrolytes (open symbols) have a significantly higher static dielectric constant, *ε_e_* > *ε_s_*. This is expected and can be explained through a strong increase in the polarization of the polymer electrolytes. Another interesting observation is the chemistry dependence of the salt concentration on the static dielectric constants. PECH/LiTFSI electrolytes have *ε_e_* ∼ 20, and PBO/LiTFSI has *ε_e_* ∼ 10, which change only slightly with the salt concentration and temperature. In contrast, *ε_e_* of PPO/LiTFSI varies significantly with the salt concentration.

**Figure 8:**
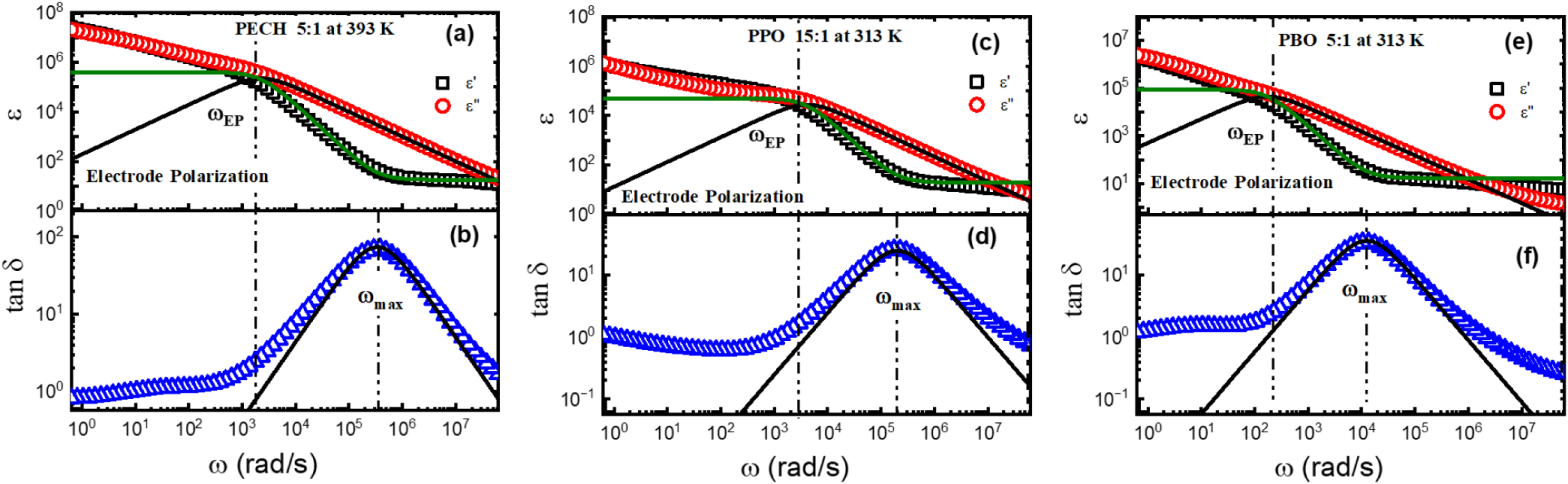
Dielectric spectrum ε′(ω), ε″(ω), and tan δ­(ω) of (a-b) PECH 5:1 at 393 K, (c-d) PPO 15:1 at 313 K, and (e-f) PBO 5:1 at 313 K. Here, ω_EP_ is the characteristic angular frequency of the electrode polarization and ω_max_ is the peak frequency of tan δ­(ω).

**Figure 9:**
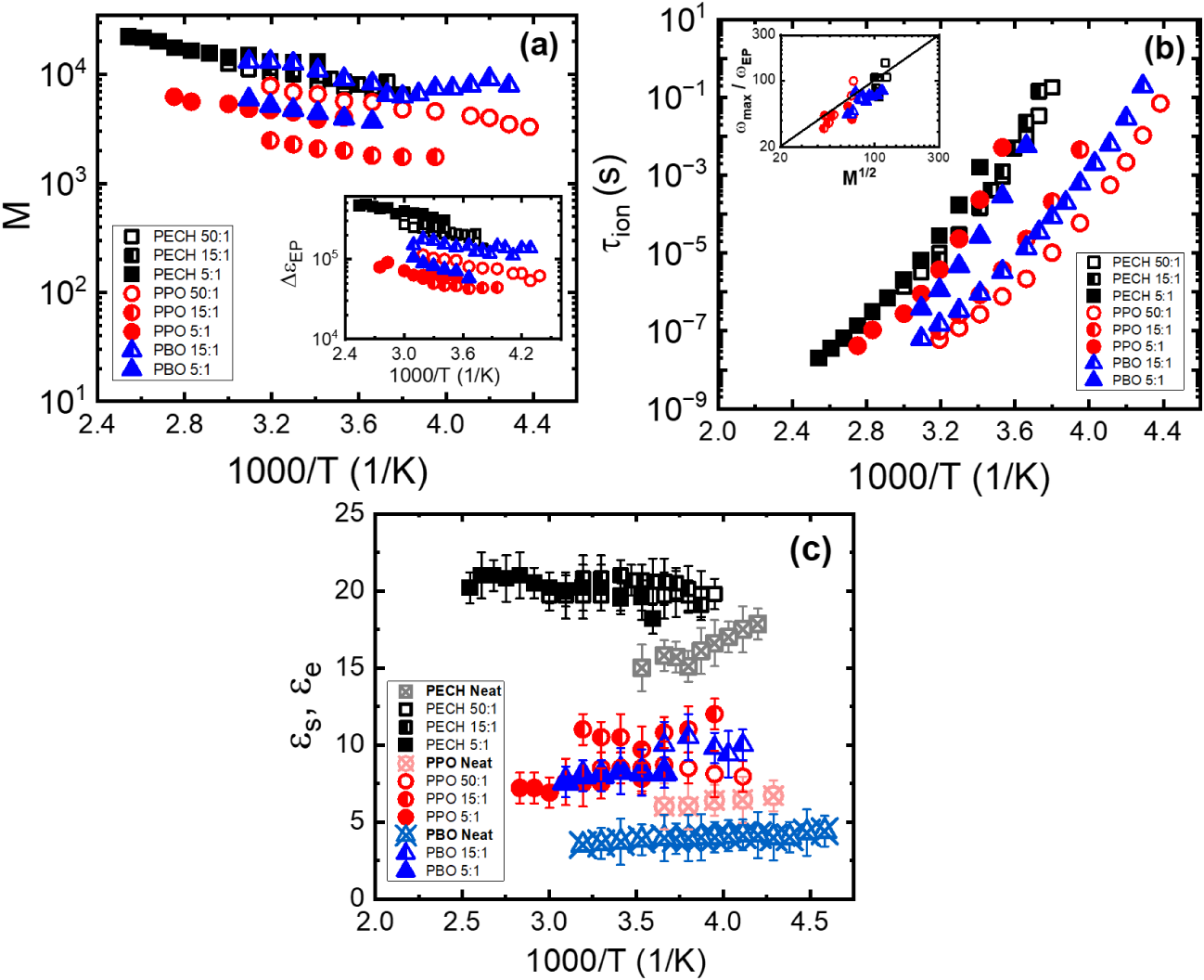
Temperature dependence of (a) *M*, (b) τ_ion_, and (c) dielectric constant of the neat polymer ε_*s*_ (filled symbols) and the polymer electrolytes ε_*e*_ (open symbols). The inset of (a) shows the temperature dependence of Δε_EP_, and the inset of (b) shows the $\frac{\omega_{\max}}{\omega_{\mathrm{EP}}}$ over $M^{\frac{1}{2}}$ of the polymer electrolytes.

#### 3.3.3 Polymer Dynamics and Ionic Conductivity

Dielectric properties of polymer electrolytes at frequencies higher than the EP contain information about polymer dynamics. Specifically, the dielectric spectra can be analyzed with Havriliak–Negami (HN) functions, as expressed in Equation 3:

3 $$\varepsilon^{*}\left(\omega \right)=\varepsilon_{\infty }+\underset{j}{\sum }\frac{\Delta \varepsilon_{j}}{{\left(1+{\left(i\omega \tau_{\mathrm{HN},j}\right)}^{\beta_{j}}\right)}^{\gamma_{j}}}+\frac{\sigma_{\mathrm{DC}}^{\mathrm{HN}}}{i\varepsilon_{0}\omega }+A\omega^{-n}$$

where *ε*_∞_ is the dielectric permittivity at infinitely high frequencies, *τ*_HN,*j*_, *β_j_*, and *γ_j_* are the HN relaxation time, the asymmetric stretching, and the symmetric stretching parameters of the *j* ^th^ dielectric relaxation, respectively, $\sigma_{\mathrm{DC}}^{\mathrm{HN}}$ is the DC conductivity from the HN analysis, and A and *n* are two constants describing the Jonscher process.^65,66^ The average relaxation time of the $j^{\mathrm{th}}$ process can be estimated from the *τ*_HN,*j*_ through $\tau_{j}=\tau_{\mathrm{HN},j}{[\sin\left(\frac{\beta_{j}\pi }{2+2\gamma_{j}}\right)]}^{-1/\beta_{j}}{[\sin\left(\frac{\beta_{j}\gamma_{j}\pi }{2+2\gamma_{j}}\right)]}^{1/\beta_{j}}$ . The $\frac{\sigma_{\mathrm{DC}}^{\mathrm{HN}}}{i\varepsilon_{0}\omega }$ and *Aω* ^–^ *^n^* describe the dielectric responses of the crossover between the EP and the polymer dynamics.

Figure 10 presents the dielectric loss permittivity, *ε*″(*ω*), the derivative spectra $\varepsilon_{\mathrm{der}}^{’}\left(\omega \right)$ , and the real part of the complex conductivity, *σ*′(*ω*), of PECH/LiTFSI at [O]:[Li] = 50:1 at *T* = 283 K (Figure 10 a–c), PPO/LiTFSI at [O]:[Li] = 50:1 at *T* = 253 K (Figure 10 d–f), and PBO/LiTFSI at [O]:[Li] = 15:1 at *T* = 283 K (Figure 10g-i). The symbols are experimental results; the dashed, dash-dotted, and dotted lines are the fits to HN functions, and the solid lines are the sum of the fit that include the DC conductivity and the EP contribution. The high DC conductivity of polymer electrolytes shields the structural relaxation process in the *ε*″(*ω*). On the other hand, $\varepsilon_{\mathrm{der}}^{’}\left(\omega \right)$ does not contain the contribution of the DC conductivity and gives clear signatures of the structural relaxation (the blue dash-dotted lines with a characteristic peak frequency of *ω*_*α*_) (Figure 10 b, e, h).^67^ The green dotted lines at the high frequency represent secondary relaxations with a characteristic frequency of *ω_β_* (see Figure S3). At the same time, one can directly obtain the DC conductivity, *σ*_DC_, from the plateau in the real part of the conductivity spectra, *σ*′(*ω*) (Figure 10c, f, i)^68^

**Figure 10:**
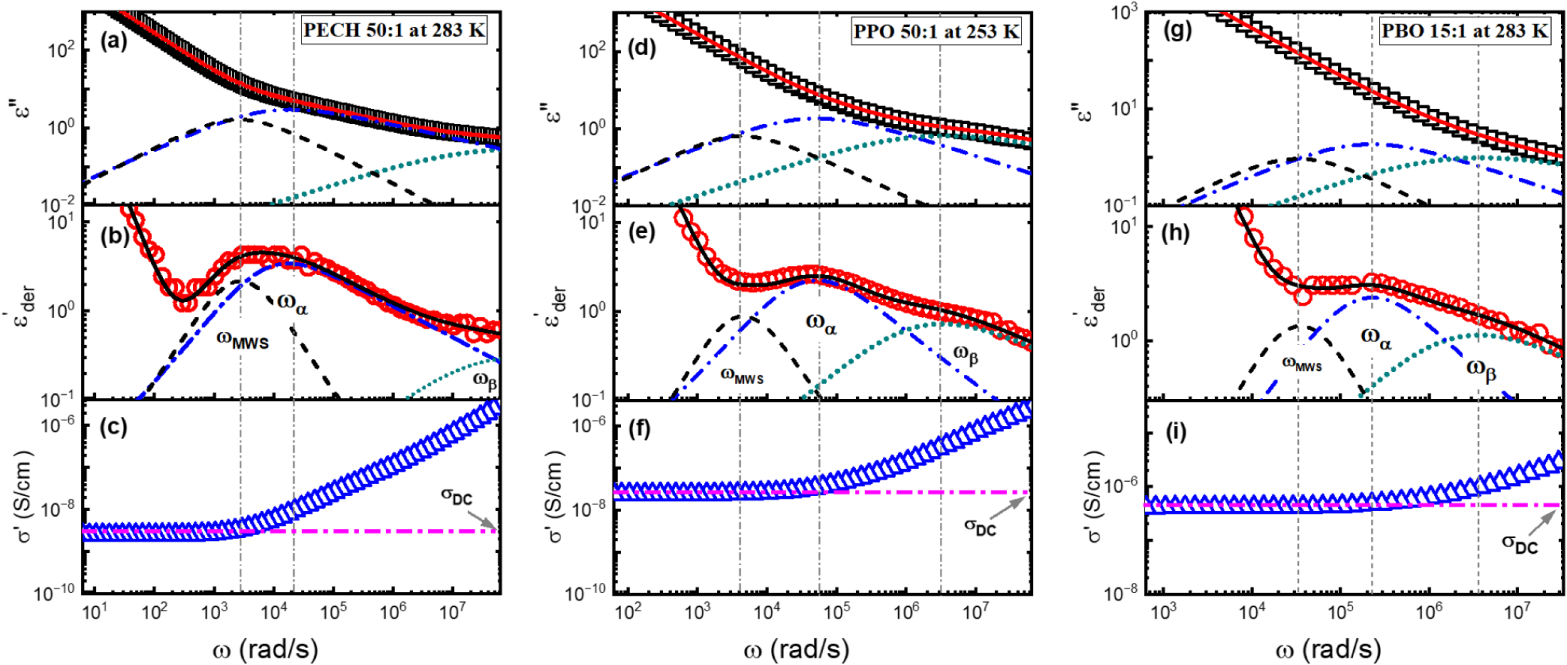
Dielectric spectrum of (a–c) PECH/LiTFSI with [O]:[Li] = 50:1 at 283 K, (d–f) PPO/LiTFSI with [O]:[Li] = 50:1 at 253 K, and (g–i) PBO/LiTFSI with [O]:[Li] = 15:1 at 283 K. The dashed lines, the dash-dotted lines, and the dotted lines are the fits to the MWS, the structural relaxation, and the secondary relaxation processes, with ω_MWS_, ω_α_, and ω_β_ being the characteristic relaxation rates, respectively. The solid lines represent a sum of all relaxation processes, including the DC conductivity and electrode polarization contributions.

Figure 11a–c presents the temperature dependence of *τ_α_* of the polymer electrolytes and the neat polymers from the HN analyses. The solid lines are the corresponding fit to the VFT equation, $\tau_{\alpha }\left(T\right)=\tau_{0}\exp\left(\frac{B}{T-T_{0}}\right)$ , where *τ*_0_ and *B* are constants and *T*_0_ is the Vogel temperature. Extrapolating to *τ_α_* = 100 s, one can define a dynamic glass transition temperature from BDS measurements, $T_{g}^{\mathrm{BDS}}$ . Experimentally, $T_{g}^{\mathrm{BDS}}\approx T_{g}^{\mathrm{DSC}}$ holds (see Table 1), confirming the assignment of the dielectric structural relaxation. A large influence of salt on *τ_α_* have been observed for all electrolytes at low temperatures. However, at high temperatures, the slowing down of the polymer electrolytes *τ_α_* with increasing salt loading is significantly reduced. This is quite intriguing, as a higher temperature should lead to higher salt solvation and one would expect a larger influence of ion-to-polymer dynamics. Interestingly, the dynamic fragility indexes, $m=\frac{dlog_{10}\tau_{\alpha }}{d\left(\frac{T_{g}}{T}\right)}{|}_{T=T_{g}}$ , of these polymer electrolytes vary only slightly with the salt concentration (see Table 2).

**Figure 11:**
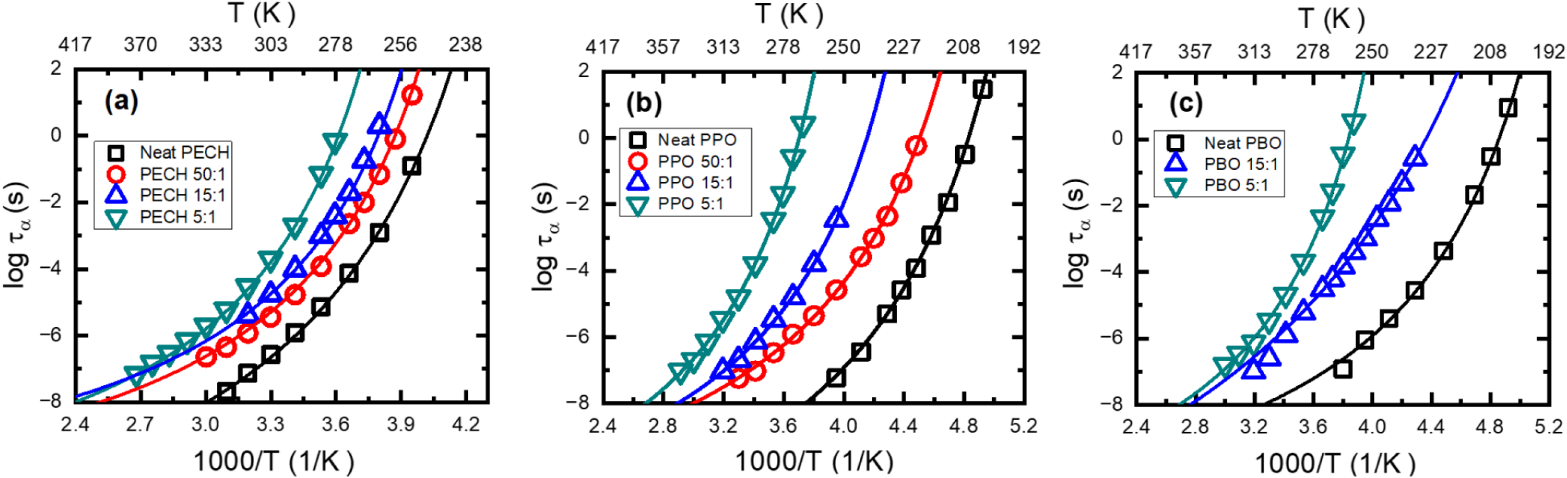
Temperature dependence of segmental relaxation time from BDS (τ_α_) for (a) PECH, (b) PPO, and (c) PBO systems. The solid lines are VFT fit results.

**Table 2:** Vogel–Fulcher–Tammann Fit Parameters

	PECH/LiTFSI				PPO/LiTFSI				PBO/LiTFSI			
**[O]:[Li]**	**τ**_**0**_ (**s**)	***B* (K)**	***T***_**0**_ **(K)**	***m***	**τ**_**0**_ (**s**)	***B* (K)**	***T***_**0**_ **(K)**	***m***	**τ**_**0**_ (**s**)	***B* (K)**	***T***_**0**_ **(K)**	***m***
**Neat**	1.7 × 10^–13^	1478	199	82	6.3 × 10^–14^	1176	168	91	1.0 × 10^–11^	971	168	80
**50:1**	2.3 × 10^–11^	1086	214	84	5.6 × 10^–12^	1162	177	75	-----	-----	-----	--
**15:1**	2.8 × 10^–11^	1133	218	82	3.0 × 10^–12^	1227	194	80	1.8 × 10^–13^	2317	150	59
**5:1**	2.1 × 10^–11^	1191	229	83	1.4 × 10^–12^	1346	221	86	4.2 × 10^–12^	1229	214	85

Figure 12a summarizes *σ*_DC_ from the plateau of the *σ*′(*ω*) (the open symbols), which follows the VFT temperature dependence. To compare the analyses of DC conductivities from different methods, Figure 12a includes $\sigma_{\mathrm{DC}}^{\mathrm{HN}}$ from the HN function fit (the red solid line) and $\sigma_{\mathrm{DC}}^{\mathrm{BNN}}$ from the BNN relation with *C* = 1.35 × 10^–4^ (the black dashed line) for one representative sample, PPO/LiTFSI at [O]:[Li] = 50:1. Excellent agreement is observed, highlighting the consistency of different analyses of DC conductivity. *σ*_DC_ of standard PEO/LiTFSI with a molecular weight of 8 kg/mol and [O]:[Li] = 50:1 and 5:1 are also presented in Figure 12a for comparison. Figure 12b summarizes *σ*_DC_ over the salt volume fraction *ϕ*(%) of polymer electrolytes at 293 K (open symbols) and 313 K (filled symbols). In general, PECH/LiTFSI electrolytes give the lowest DC conductivity among the three, despite neat PECH having the largest static dielectric constant. For a given polyether, a higher salt concentration gives a higher *T*_g_ and a lower DC conductivity. Interestingly, *σ*_DC_ of PPO/LiTFSI and PBO/LiTFSI are comparable at both [O]:[Li] = 15:1 and 5:1, although the repeating unit of PPO has a slightly larger volume dipolar density than that of PBO. Among all polymer electrolytes, the highest DC conductivity is from PPO/LiTFSI at [O]:[Li] = 50:1 with *σ*_DC_ ∼ 10^–5^ S/cm at room temperature, which is slightly smaller than that of PEO/LiTFSI electrolytes at a similar salt loading. Overall, these results suggest an absence of a direct correlation between the polymer static dielectric constant, the glass transition temperature, and the DC conductivity of these polyether electrolytes.

**Figure 12:**
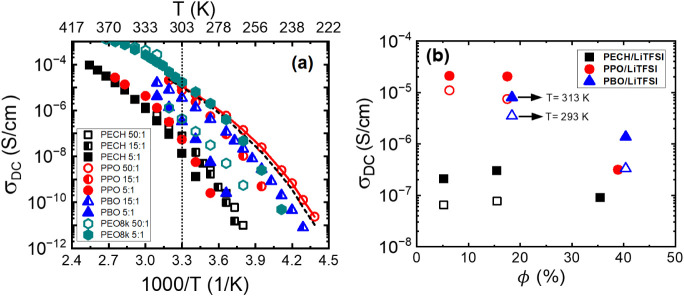
(a) Temperature dependence of σ_DC_ from the plateau of the σ′(ω) (the open symbols). The solid line shows DC conductivity from the HN function fit $\left(\sigma_{\mathrm{DC}}^{\mathrm{HN}}\right)$ , and the dashed lines show $\sigma_{\mathrm{DC}}^{\mathrm{BNN}}$ of the BNN equation with *C* = 1.35 × 10^–4^ for PPO/LiTFSI at [O]:[Li] = 50:1. We also measured the σ_DC_ of PEO/LiTFSI with a molecular weight of 8 kg/mol and [O]:[Li] of 50:1 and 5:1 for comparison. (b) σ_DC_ vs salt weight fraction ϕ(%) at 293 K (open symbols) and 313 K (filled symbols).

#### 3.3.4 Apparent Activation Energy for Free-Ion Generation

Combining the DC conductivity, *σ*_DC_, and the ion mobility, μ=eL^2^/(4M^2^τ_ion_k_B_T), one can estimate the free-ion concentration, *n_f_*, through the Nernst–Einstein relation, *n_f_* = *σ*_DC_/(*qμ*).^54,60^ The temperature dependence of *n_f_* (*T*) can then provide an estimate of the activation energy for the free-ion generation. Figure 13 gives the temperature dependence of the free-ion fraction *n_f_*/*n*_tot_, where *n*_tot_ is total ion concentration, assuming 100% salt dissociation. Several important notes can be made: (i) For all samples, the free-ion number density decreases with temperature. An exceedingly small fraction of the total ions are free at the testing temperatures, *n_f_/n*_tot_ ∼ 0.003%–1%, in contrast to the significantly higher static dielectric constant of the polymer electrolyte than the neat polymers (Figure 9c). This observation indicates the presence of a large amount of trapped Li^+^, which has been revealed by computer simulations.^25,69,70^ (ii) For a given matrix polymer, electrolytes with a lower salt concentration end up with a lower total amount of free ions and higher free-ion fractions. (iii) An Arrhenius temperature dependence of $n_{f}/n_{\mathrm{tot}}=A\exp\left(\frac{-E_{a}}{k_{B}T}\right)$ has been observed, implying that free-ion generation is an activation process, where *A* is a constant prefactor and *E_a_* is the activation energy for free-ion generation. *E_a_* ≈ 6 kJ/mol is observed for all polymer electrolytes, regardless of the static dielectric constant of the parent polyether. Note that a similar activation energy for free-ion generation has been observed for other polymer electrolytes with LiTFSI, indicating that *E_a_* ≈ 6 kJ/mol as an intrinsic molecular parameter describing the free-ion generation of LiTFSI in polyethers.^60^ We note that FT-IR has been employed to characterize the ion solvation state, which provides a qualitative measure of the ion solvation status.^51^ Since the solvated ion stays mostly in the trapped state in polymer electrolytes, it is challenging to obtain the free-ion concentrations from FT-IR measurements that correlate directly with the DC conductivity.

**Figure 13:**
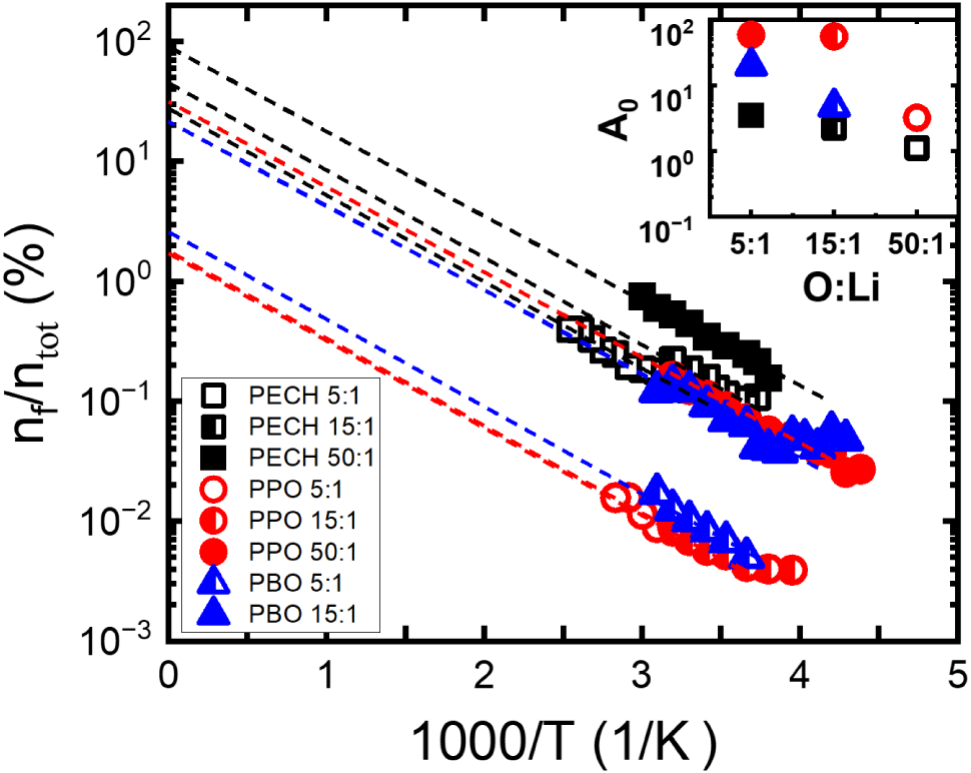
Temperature dependence of the free-ion fraction $\frac{n_{f}}{n_{\mathrm{tot}}}\left(\%\right)$ , evaluated from the electrode polarization analysis, where *n*_tot_ is the free-ion concentration limit at complete dissociation. Solid lines denote Arrhenius fits $n_{f}=n_{0}\exp\left(\frac{-E_{a}}{k_{B}T}\right)$ , where *n*_0_ is the infinite high-temperature limit of *n*_*f*_ (*n*_tot_ ≠ *n*_0_) and the inset shows *A* = *n*_tot_ */n*_0_ for different ion concentrations.

On the other hand, a linear extrapolation of the In­(*n_f_*/*n*_tot_) at infinitely high temperatures does not lead to a generation of 100% free ions, i.e., $({\frac{n_{f}}{n_{\mathrm{tot}}})|}_{T\to \infty }\neq 1$ . This phenomenon has been observed before both in polymer electrolytes and in polymerized ionic liquids and has been attributed to an underestimation of the free-ion concentration from the analysis of the McDonald model.^60^ By introducing a correction parameter *A*_0_ (the inset of Figure 13) that gives $\frac{n_{f}}{n_{\mathrm{tot}}/A_{0}}=1$ at $\left(\frac{1000}{T}\right)\to 0$ (or $A_{0}={\frac{n_{\mathrm{tot}}}{n_{f}})|}_{T\to \infty })$ , one can obtain an effective free-ion concentration, $n_{f}^{*}=A_{0}n_{f}$ . As previous experiments show, such a correction can lead to a correct estimate of the ion diffusion coefficient, *D*, through the Nernst–Einstein relation, $D=\frac{\sigma_{\mathrm{DC}}k_{B}T}{q^{2}n_{f}^{*}}$ , that agrees well with the nuclear magnetic resonance spectroscopy (NMR) measurements for ion diffusion over wide temperatures.^54,60^ In this study, we focus on the apparent activation energies for free-ion generation that remain the same for both *n_f_* and $n_{f}^{*}$ .

### 3.4 Rheology

To understand the effect of salt on dynamics slower than the structural relaxation, we turn to linear rheology, which can be compared with BDS. Figure 14 presents the storage modulus, *G’*(*ω*), and the loss modulus, *G*’’(*ω*), of neat PECH (Figure 14a), PECH/LiTFSI at [O]:[Li] = 50:1 (Figure 14b), PECH/LiTFSI at [O]:[Li] = 15:1 (Figure 14c), and PECH/LiTFSI at [O]:[Li] = 5:1 (Figure 14d) at the reference temperature of *T*_ref_ = 273 K. The insets of Figure 14a–d give the corresponding Van Gurp-Palmen plots to demonstrate the rheological simplicity of these samples.^71^ No vertical shifts are needed to construct the linear viscoelastic master curves. Normalizing the dynamics frequency to the characteristic peak of the structural relaxation, *ω_α_*, Figure 14e,f provides a comparison of PECH/LiTFSI at different salt concentrations. The PECH/LiTFSI electrolytes have a broadened structural relaxation peak compared with the neat PECH, and there is a shrinkage in the rubbery plateau. Interestingly, the salt affects little the separation between terminal relaxation and the structural relaxation (Figure 14f). These results suggest that the salt affects the terminal dynamics of the polymer electrolytes primarily through a slowing down of the segmental motion. Similar analyses have been conducted for PPO/LiTFSI (see Figure S4). From the characteristic peak of *G*’’(*ω*) at the high frequencies or the crossover between *G’*(*ω*) and *G*’’(*ω*), one can estimate important characteristic time scales: the characteristic relaxation rate for structural relaxation, *ω_α_*, at the high frequency peak of the *G*’’(*ω*), the relaxation rate for entanglement strand, *ω*_*e*_, at the intermediate crossover between *G’*(*ω*) and *G*’’(*ω*), and the characteristic relaxation rate for terminal flow, *ω_f_* (see Figure 14a). From these characteristic relaxation rates, one can obtain the structural relaxation time, *τ_α_* ≈ 1/*ω_α_*, the apparent entanglement time, *τ*_e_ ≈ 1/*ω_e_*, and the terminal relaxation time, *τ_f_* ≈ 1/*ω_f_*.

**Figure 14:**
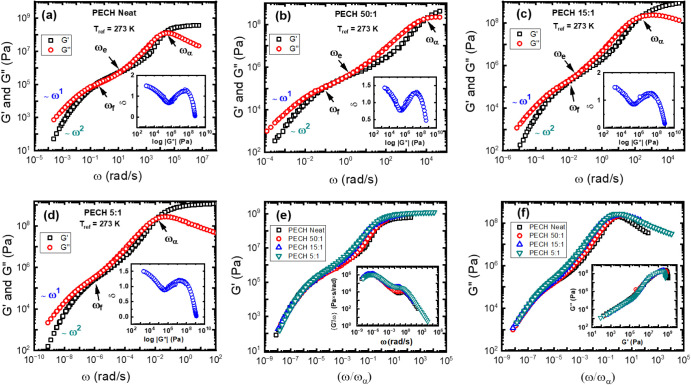
Rheology data of (a) PECH neat, (b) PECH 50:1, (c) PECH 15:1, and (d) PECH 5:1. The insets in (a–d) are Van Gurp–Palmen plots between the phase angle δ (in radians) and the log_10_| *G* *| for all the PECH samples. (e) The storage modulus and (f) the loss modulus for all the PECH samples over ω/ω_α_. The inset in (e) is *G*'/ω over ω, and that in (f) is *G*″ over *G*’, for the same samples.

#### 3.4.1 Structural Relaxation

Figure 15a,c presents representative comparisons between the dielectric loss spectra, ε"(ω), and derivative spectra, $\varepsilon_{\mathrm{der}}^{’}\left(\omega \right)$ , of the neat PECH and PECH/LiTFSI at [O]:[Li] = 5:1 and *T* = 273 K, and Figure 15b,d presents the linear viscoelastic master curve of the corresponding sample with *T*_ref_ = 273 K. The structural relaxation of the neat PECH is well-resolved in both *G*’’(*ω*) and $\varepsilon_{\mathrm{der}}^{’}\left(\omega \right)$ (Figure 15a). The characteristic angular frequency of the structural relaxation peak from BDS agrees well with the loss peak of *G*’’(*ω*) (Figure 15b). The results of Figure 15a,b indicate comparable structural relaxation times from dielectric measurements and rheology for neat PECH. At the same time, the DC conductivity of the neat PECH shields the dielectric normal modes of PECH, and linear rheology reveals that the terminal mode of neat PECH is around five decades slower than its structural relaxation.

**Figure 15:**
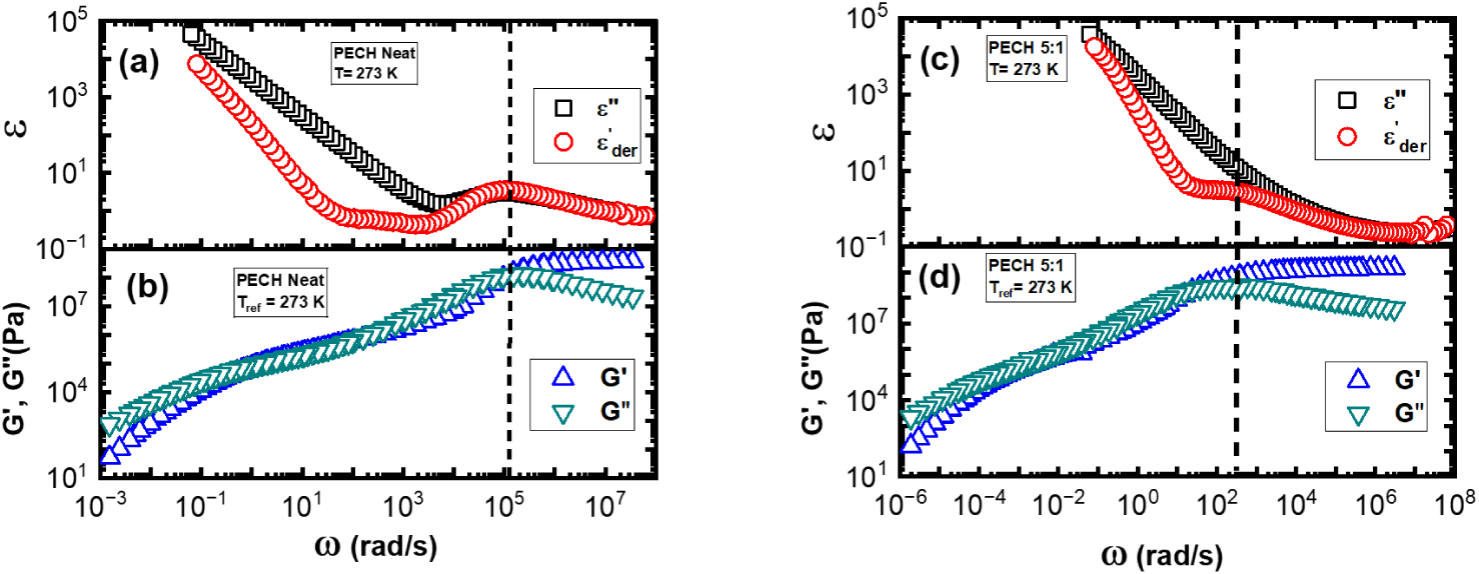
Comparison between dielectric measurements ε″(ω), ε_der_’(ω) and linear viscoelastic spectra *G*’(ω), *G*″(ω) for (a, b) neat PECH and (c, d) PECH/LiTFSI at [O]:[Li] = 5:1, both at *T* = *T*_ref_ = 273 K.

In polymer electrolytes, a high DC conductivity shields their structural relaxation in loss permittivity, *ε*’’(*ω*), while $\varepsilon_{\mathrm{der}}^{’}\left(\omega \right)$ exhibits a relaxation process at intermediate frequencies. As shown in Figure 15c, the relaxation peak is only visible in the derivative spectra of PECH/LiTFSI with [O]:[Li] = 5:1 at *T* = 273 K. At the same time, the linear viscoelastic spectra of the PECH/LiTFSI at [O]:[Li] = 5:1 at *T* = 273 K, gives clear signatures of the structural relaxation (Figure 15d). The characteristic angular frequency of the peak of *G*’’(*ω*) agrees well with the structural relaxation peak from $\varepsilon_{\mathrm{der}}^{’}\left(\omega \right)$ . We would like to emphasize that the $T_{g}^{\mathrm{BDS}}\approx \approx T_{g}^{\mathrm{Rhg}}\approx T_{g}^{\mathrm{DSC}}$ holds (Table 1) as well. Therefore, these analyses further support the assignment of structural relaxation in dielectric measurements.

#### 3.4.2 Chain Dynamics and Entanglement

Rheology provides characterizations of the chain dynamics and polymer entanglement. As shown in Figure 14a, the neat PECH has *ω*_*e*_ ≈ 17 rad/s and ω_f_ ≈ 0.2 rad/s at the crossover between *G*’(*ω*) and *G*’’(*ω*) at the intermediate and low frequencies, respectively. *ω_e_*/*ω_f_* ≈ 85 provides a rough estimate of the length of the rubbery plateau and corresponds to the number of entanglements per chain of *Z* ≈ 3.^72^ From the linear rheology, one can also obtain the plateau modulus of neat PECH, *G_N_* = 0.32 MPa, which gives the molecular weight between entanglements, *M_e_* ≈ *ρRT*/*G_N_* = 10.6 kg/mol. The corresponding molecular weight of neat PECH polymer is *M*_w_ ≈ 32 kg/mol, which agrees well with the size exclusion chromatography (SEC) measurements (*M*_w_ ≈ 30.7 kg/mol).^47^ Including LiTFSI into the PECH affects *ω_α_*, *ω_e_*, and *ω_f_*. In particular, the plateau length shrinks from ∼85 of the neat PECH to ∼16 at [O]:[Li] = 50:1, ∼4 at [O]:[Li] = 15:1, and completely diminishes the rubbery plateau at [O]:[Li] = 5:1. At the same time, normalizing the frequencies of the storage and loss moduli with *ω_α_*, one can find little changes in *ω*_*α*_/*ω_f_* with salt concentration (Figure 14e,f) and negligible changes in the plateau modulus. Similar observations have also been found in PPO/LiTFSI (Figure S4), implying a general feature of the influence of salt on the rheology of the polyether electrolytes. This lack of modulus reduction rules out the mechanism of the solvated salt serving as a solvent to dilute the entanglement network since the entanglement network dilution would lead to a strong reduction in the plateau modulus.^73,74^ Furthermore, given the attractive polymer–Li^+^ interactions, it is also quite intriguing to see the shrinkage in the rubbery plateau and the almost constant *τ_f_*/*τ_α_* of polymer electrolytes with different salt concentrations. These results highlight a complex interplay between polymer-ion interactions and entanglement dynamics.

### 3.5 Comparison Between Dielectric Spectroscopy and Rheology

Both BDS and rheology provide measurements of the dynamics of polymer electrolytes. However, BDS captures the local dipole reorientation dynamics, while rheology captures molecular dissipation at various length scales. For polymer systems with network structures, the difference in the temperature dependence of the dynamics shift factors from rheology, $a_{T}^{R}$ , and from BDS, $a_{T}^{\mathrm{BDS}}=\tau_{\alpha }\left(T\right)/\tau_{\alpha }\left(T_{\mathrm{ref}}\right)$ , provide important information of the network dynamics in ion-containing polymers.^75,76^ Figure 16 presents the $a_{T}^{R}$ and $a_{T}^{\mathrm{BDS}}$ of the three types of polymer electrolytes. Several interesting features are worth noting: (i) $a_{T}^{R}\approx a_{T}^{\mathrm{BDS}}$ holds for neat PECH and neat PPO (Figure S5), indicating the absence of decoupling between the segmental dynamics and chain dynamics in the neat polymer.^77^ (ii) Despite the similar temperature dependence of $a_{T}^{R}$ and $a_{T}^{\mathrm{BDS}}$ of the neat polymer, $a_{T}^{R}$ and $a_{T}^{\mathrm{BDS}}$ of polymer electrolytes exhibit different temperature dependence close to *T*_g_ (Figure 16). The different temperature dependences of $a_{T}^{R}$ and $a_{T}^{\mathrm{BDS}}$ at low temperatures suggest the influence of salt on dynamic modes at length scales longer than the structural relaxation, such as the emergence of a transient salt-induced polymer network. In particular, at times shorter than the transient network dissociation, *τ*_dis_, network dissociation has yet to take place and the ion serves as cross-link points, which also contribute to the shift in *T*_g_. At $t\gg \tau_{\mathrm{dis}}$ , $a_{T}^{R}∼a_{T}^{\mathrm{BDS}}$ is observed for all polymer electrolytes. The negligible influence of the terminal flow of polymer electrolytes thus suggests $\tau_{f}\gg \tau_{\mathrm{dis}}$ . Note that the emergence of a salt-induced transient network in polymer electrolytes has been proposed before.^78^ The characteristics of the network dynamics have also been discussed recently in the context of associative polymers and ionomers,^53,79^ where different temperature dependences of $a_{T}^{R}$ and $a_{T}^{\mathrm{BDS}}$ have been observed at high temperatures to estimate the apparent activation energy of polymer-ion dissociation.^76^ Our recent experiments also show little changes in their linear viscoelastic spectra when sticky dissociation is much faster than the terminal relaxation time, $\tau_{f}\gg \tau_{\mathrm{dis}}$ , supporting the above-proposed understanding.^80^

**Figure 16:**
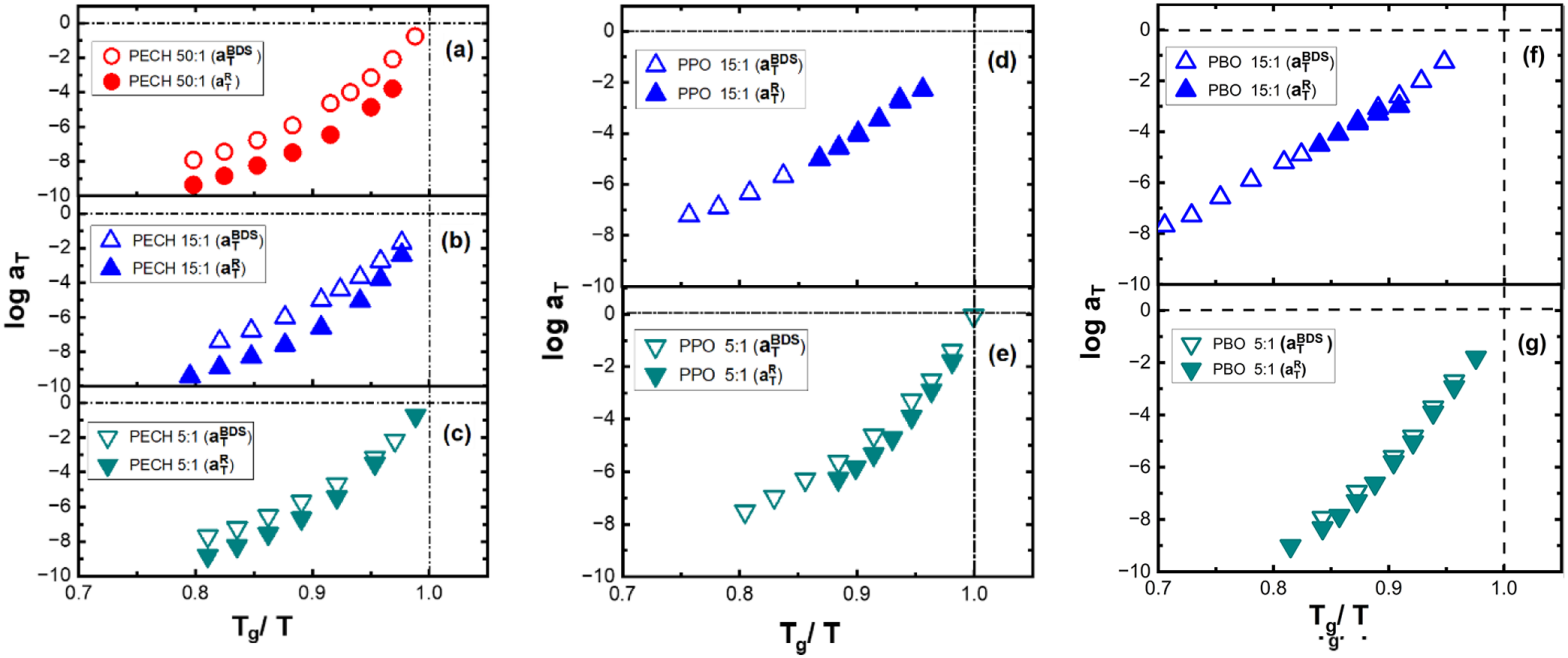
Comparison between $a_{T}^{\mathrm{BDS}}$ (the open symbols) and $a_{T}^{R}$ (the filled symbols) of (a–c) PECH/LiTFSI, (d, e) PPO/LiTFSI, and (f, g) PBO/LiTFSI, over *T*_g_/*T*.

Plotting $\ln\left(a_{T}^{R}/a_{T}^{\mathrm{BDS}}\right)$ vs 1000/*T* yields linear lines at low temperatures (Figure 17), indicating an Arrhenius temperature dependence of $a_{T}^{R}/a_{T}^{\mathrm{BDS}}$ at these temperatures. Therefore, a strong connection between *τ_α_* and *τ*_dis_ can be envisioned. The Arrhenius temperature dependence of $a_{T}^{R}/a_{T}^{\mathrm{BDS}}$ further indicates an Arrhenius relation between *τ*_dis_ and *τ_α_*: $\tau_{\mathrm{dis}}\left(T\right)\approx \tau_{\alpha }\left(T\right)\exp\left(\frac{\Delta E_{n}}{RT}\right)$ with Δ*E_n_* being the apparent activation energy. Similar observations have been observed in ionomers^76^ and polymer nanocomposites.^75,81^ From the slope of the linear dependence of $\ln\left(a_{T}^{R}/a_{T}^{\mathrm{BDS}}\right)$ vs 1000/T , one can estimate 62 kJ/mol for both PECH/LiTFSI at [O]:[Li] = 5:1 and 15:1, 41 kJ/mol for PPO/LiTFSI at [O]:[Li] = 5:1, and 16 kJ/mol for PBO/LiTFSI at [O]:[Li] = 5:1. For a given type of polymer electrolyte, negligible variations of Δ*E_n_* have been observed with the salt concentrations, implying the polymer–ion interaction dominating the Δ*E_n_*. In particular, the ion interaction with neighboring chains is the major source of network formation (Figure 18). Furthermore, Δ*E_n_* varies quite noticeably for different types of polymer electrolytes, Δ*E_n_* (PECH/LiTFSI) > Δ*E_n_* (PPO/LiTFSI) > Δ*E_n_* (PBO/LiTFSI), which are in line with the static dielectric constants of the matrix polymer.

**Figure 17:**
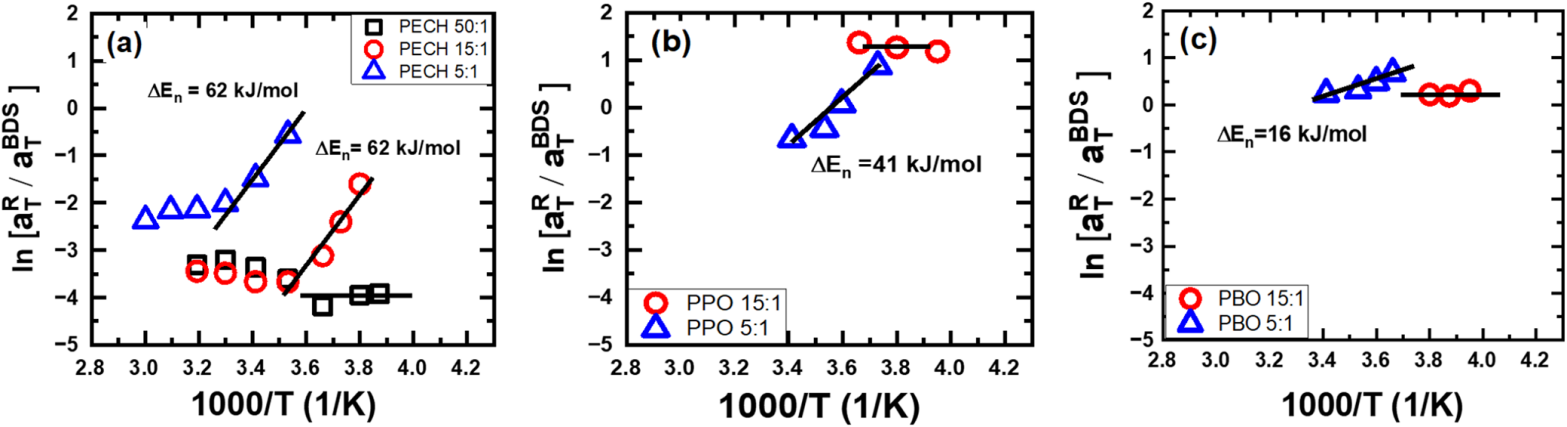
The $\ln\left(a_{T}^{R}/a_{T}^{\mathrm{BDS}}\right)$ vs 1000/*T* for (a) PECH/LiTFSI, (b) PPO/LiTFSI, and (c) PBO/LiTFSI.

**Figure 18:**
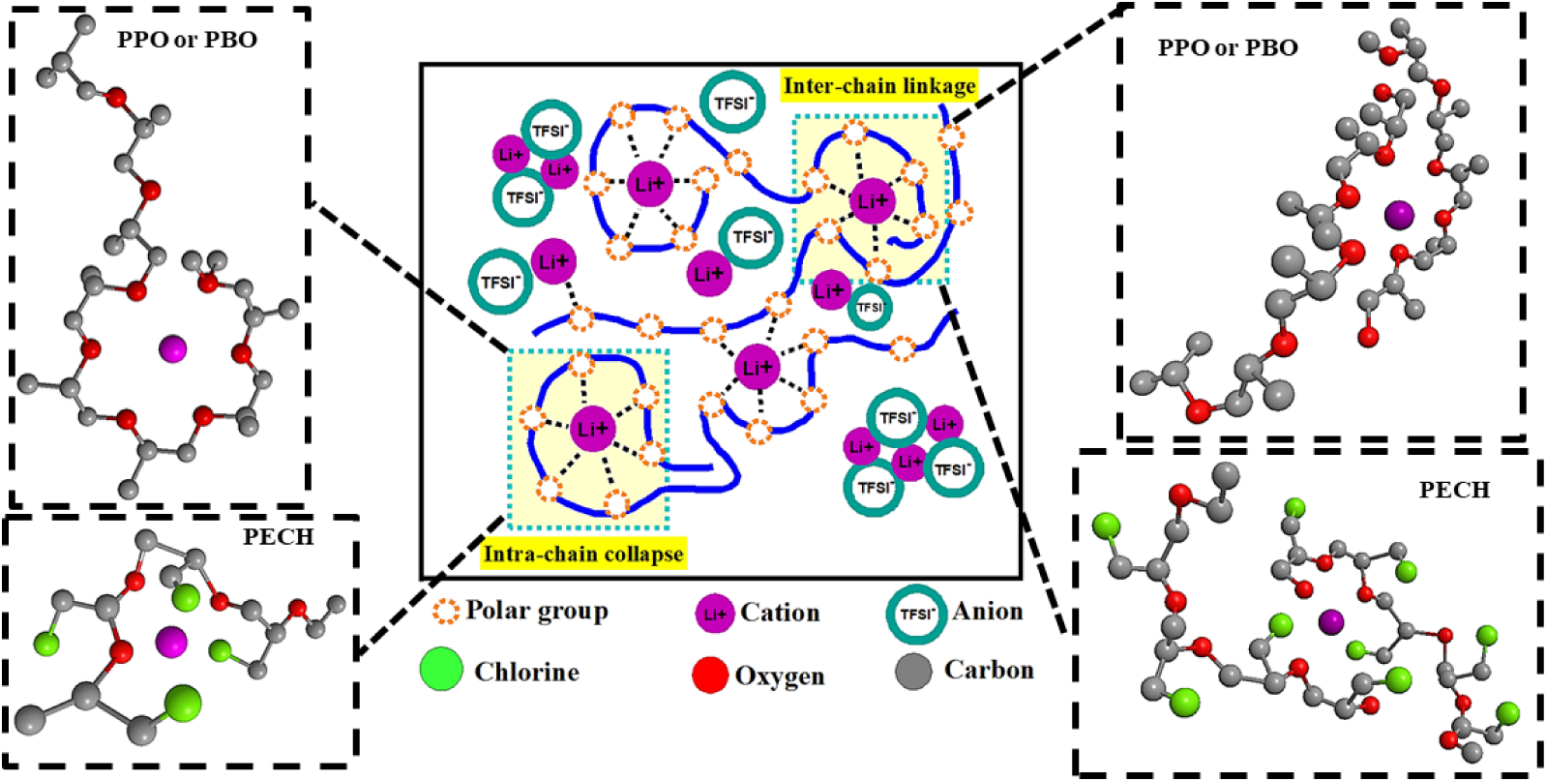
Schematic illustration of the intrachain polymer-ion complex that modifies the local chain conformation, and the interchain polymer-ion complex that links neighboring chains. The hydrogen atom is omitted for simplicity in the 3D chemical drawing to better present the chain conformation surrounding the Li^+^.

### 3.6 The Ionic Conductivity

The above analyses provide *σ*_DC_ and *τ_α_* of polymer electrolytes, enabling further investigation of the relationship between the ionic conductivity and polymer dynamics. Figures 19 plots log_10_Λ vs *–*log_10_ *τ_α_*, where $\Lambda =\frac{\sigma_{\mathrm{DC}}}{n_{\mathrm{tot}}}$ is the molar conductivity.^82^ log_10_Λ ∼ −log_10_ *τ_α_* with a slope of one has been observed, indicating a strong coupling between Λ and *τ_α_* for these polyether electrolytes. These observations are consistent with the previous discussion on the segmental dynamics dictating the ion transport in polyethers.^20,78,82^

**Figure 19:**
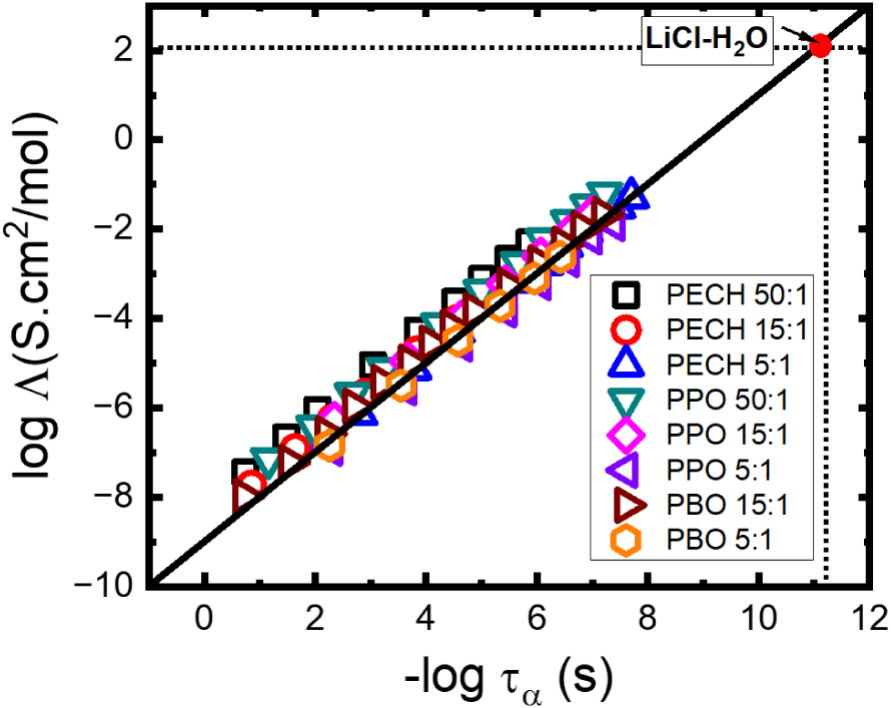
logΛ vs $-\log\tau_{\alpha }$ of all of the polymer electrolyte systems. The result for LiCl–H_2_O is added as a reference point.

### 3.7 Effect of Polymer Polarity

The combination of BDS, rheology, and DSC reveals several interesting features of the polymer polarities on the dynamics, the *T_g_*, and the room-temperature ionic conductivity of the polyether electrolytes. The static dielectric constants of the neat polymers, *ε_s_*, have the following order: PECH > PPO > PBO. However, PPO/LiTFSI has the largest shifts in *T*_g_, followed by PBO/LiTFSI, and PECH/LiTFSI (Figure 4). Thus, the salt-induced *T*_g_ shifts do not seem to correlate with *ε_s_*. At the same time, the largest room-temperature DC conductivity is PPO/LiTFSI at [O]:[Li] = 50:1, which has an intermediate dielectric constant close to PEO. These results seem to support recent computer simulations on a balanced dielectric constant of polyethers comparable to PEO that exhibit the highest room-temperature ionic conductivity.^25,27^

In addition, the combined analyses of rheology and BDS reveal several new interesting features of dynamics slower than the structural relaxation of these polymer electrolytes: (i) The temperature dependence of the dynamics shift factors from rheology, $a_{T}^{R}$ , differ from that of BDS, $a_{T}^{\mathrm{BDS}}$ , by an Arrhenius factor at low temperatures. These observations suggest the presence of a transient salt-induced polymer network with an elementary dynamics mode, *τ*_dis_(*T*), that connects to the structural relaxation, $\tau_{\mathrm{dis}}\left(T\right)\approx \tau_{\alpha }\left(T\right)\exp\left(\frac{\Delta E_{n}}{RT}\right)$ . (ii) Despite *τ*_dis_(*T*) > *τ_α_*, the intermediate dynamics slowing down does not lead to Sticky-Rouse types of dynamics.^83^ Instead, the linear rheology of these polymer electrolytes reveals an apparent disentanglement in these polyether electrolytes along with negligible reduction in the plateau modulus. (iii) The values of Δ*E_n_*, following PECH/LiTFSI > PPO/LiTFSI > PBO/LiTFSI, suggest a stronger binding to Li^+^ of the polyethers with a larger dielectric constant. Another intriguing observation is the following: the polymer electrolytes with the strongest polymer–ion binding, i.e., the PECH/LiTFSI, offer the least shift in *T*_g_.

The analyses presented above highlight the complex influence of salt solvation on the structures and dynamics of polymer electrolytes. We notice that similar rheological and dynamic changes have been observed previously in polymer nanocomposites (PNCs) with small-sized nanoparticles.^84^ In particular, the attractive polymer–nanoparticle interaction in PNCs with small-sized nanoparticles slows down the segmental dynamics and leads to a transient nanoparticle network. The nanoparticle network dissociation time, *τ_n_*, is much shorter than the terminal time of the polymer $\tau_{\alpha }<\tau_{n}\ll \tau_{f}$ . Thus, *τ_f_*/*τ_α_* is barely affected. A similar mechanism could apply here for the polymer electrolytes, where the attractive polymer–cation interaction leads to network formation and slows down *τ_α_*. As long as the time scale of the network relaxation, i.e, the *τ*_dis_ in the polymer electrolytes, is much shorter than the terminal time of the polymer, *τ_f_*/*τ_α_* should remain almost constant, as observed in the rheology of PNCs with small-sized nanoparticles. This physical picture can also explain the shrinkage in the apparent rubbery plateau since *τ*_dis_ (or *τ_n_* of PNCs) can slow down *τ_e_* significantly due to their comparable times. As *τ*_dis_ is controlled by the polymer–ion interaction, it is expected that Δ*E_n_* correlates with the static dielectric constants of the matrix polymer. From this perspective, the analogy with PNCs with small nanoparticles could help to rationalize the observed strong correlation between the apparent activation energy for network relaxation and the static dielectric constant of the matrix polymer.

To explain the shift of *T*_g_ and its relationship with *ε_s_* and Δ*E_n_*, one needs additional information on the role of *ε_s_* in network formation. According to computer simulations, one Li^+^ requires ∼6 polar atoms to stabilize; these are oxygen in PEO.^85–87^ At the same time, the Li^+^ coordinating with the backbone oxygen of PEO can form either an intrachain complex or interchain complex (Figure 18). These Li^+^···O complexes have been widely observed in computer simulations and spectroscopy measurements,^88–91^ although the explicit roles of these different types of Li^+^···O complex on the macroscopic properties of polymer electrolytes remain elusive. We believe the *intrachain* and the *interchain* Li^+^···O complexes contribute differently to glass transition, rheology, and entanglement dynamics. For instance, the *interchain* Li^+^···O coordination serves as temporal cross-link points that should have a stronger impact on *T*_g_ than the intrachain Li^+^···O coordination. At the same time, the *intrachain* Li^+^···O coordination complex could convert local chain packing from a coil to a globule. The globule conformation might alter the entanglement structures of the polymer electrolytes, in analogy to the small-sized nanoparticles in the PNCs that can increase the molecular weight between entanglements.^84^ We further note that the consideration of the detailed interchain or intrachain Li^+^···O coordination provides structural support to the explanation of the rheological behavior and the entanglement dynamics of polymer electrolytes.

Furthermore, introducing polar atoms other than oxygen in the monomer can modify the local Li^+^···O coordination, leading to alterations in the interchain and intrachain coordination. For instance, the chlorine group has a comparable electronegativity with oxygen and can coordinate with Li^+^ in the PECH, which is absent in both PPO and PBO (Figure 18). Previous FT-IR spectroscopy showed active Li^+^···Cl interactions in poly­(vinyl chloride) (PVC) interacting with LiCF_3_SO_3_, implying the possibility of having an active Li^+^···Cl interaction.^92^ Therefore, both chlorine and backbone oxygen should coordinate with the same Li^+^ nearby, and they can form a five-member stable ring structure as discussed in Figure 2 and Section 3.1.

The different ion solvation structures between PECH/LiTFSI and PPO/LiTFSI or PBO/LiTFSI could lead to a profound influence on the interchain or intrachain ion solvation complex. For instance, the five-member ring structure can allow the anion to stay close to the Li^+^, resulting in a smaller population of interchain Li^+^···O coordination in PECH/LiTFSI than in PPO/LiTFSI and PBO/LiTFSI. As a result, PECH/LiTFSI enjoys a higher chance of intrachain ion solvation and exhibits the least shift in *T*_g_. We believe this offers an explanation to the observed less *T*_g_ shift of PECH/LiTFSI than PPO/LiTFSI and PBO/LiTFSI, even though PECH has the highest static dielectric constant and the largest ion solvation capability. On the other hand, PECH requires the dissociation of both the Li^+^···O coordination and Li^+^···Cl coordination of the same monomer before complete ion dissociation, which explains the much higher apparent activation energy Δ*E_n_* of PECH/LiTFSI than PPO/LiTFSI and PBO/LiTFSI. This physical picture can also explain the comparable *T*_g_ shift between PPO/LiTFSI and PBO/LiTFSI due to their comparable polar group distribution along the backbone. The slightly smaller *T*_g_ shift of PBO/LiTFSI than PPO/LiTFSI might be due to the slightly larger side group of PBO, which weakens slightly the Li^+^···O coordination in PBO compared with that in PPO, which agrees with the observed smaller Δ*E_n_* of PBO/LiTFSI than the PPO/LiTFSI. Therefore, the above observations and analyses suggest that the dynamics of polymer electrolytes are not directly correlated with the static dielectric constant of the monomer, and that the details of the Li^+^ interaction with the polar group play an essential role.

## 4 Conclusions

In conclusion, we have investigated the dynamics, glass transition, and ionic conductivity of a group of polyether electrolytes, including PBO/LiTFSI, PPO/LiTFSI, and PECH/LiTFSI, at [O]:[Li] = 50:1, 15:1, and 5:1 through a combination of X-ray scattering, FT-IR, BDS, rheology, and DSC. X-ray scattering and FT-IR demonstrate similar crown-ether-dominated ion solvation structures of PPO/LiTFSI and PBO/LiTFSI, while the presence of chloride in PECH can lead to the presence of different ion solvation structures, such as a five-member ring structure with a combination of Li^+^···Cl and Li^+^···O coordinates. DSC measurements showed polymer electrolytes exhibit either a much broader *T*_g_ step or two separated *T*_g_s, highlighting the influence of salt solvation on the glass transition or phase behavior of polymer electrolytes. Detailed analyses showed *T*_g_ of polymer electrolytes increases almost linearly with salt concentration, revealing a quantitative relationship between *T*_g_ and the salt concentration. At the same time, BDS measurements demonstrated clear slowing down in structural relaxation rates that leads to a similar dynamic glass transition temperature as the DSC. In addition, BDS also captured a reduction in DC conductivity with salt concentration from [O]:[Li] = 50:1 to [O]:[Li] = 5:1, along with a strong coupling between the molar conductivity and the structural relaxation, regardless of the phase behavior of the polymer electrolytes.

The study also reveals the intriguing influence of salt on chain dynamics and entanglement states of polymer electrolytes through linear rheology, including (i) a slowing down in entanglement dynamics *τ_e_* with respect to *τ_α_*; (ii) an almost unchanged terminal relaxation time, *τ_f_*, with respect to *τ_α_*; (iii) negligible changes in plateau modulus upon salt inclusion; and (iv) a progressive shrinkage in the rubbery plateau with salt concentration, along with complete disentanglement for all three polyether electrolytes at [O]:[Li] = 5:1. These observations reveal an interesting influence of salt on the entanglement dynamics of polymer electrolytes.

Although a higher static dielectric constant can lead to better ion solvation and a higher free-ion concentration, the apparent activation energy for salt solvation does not change with the static dielectric constant of the matrix polymer. The ionic conductivity couples strongly with the structural relaxation time for glass transition in all three types of polymer electrolytes; however, no direct correlation was found between the static dielectric constant of the neat polymer and the ionic conductivity, the glass transition, and the slowing down in segmental relaxation time. At the same time, the broadening or the appearance of two *T*_g_s in DSC measurement, the shift in *T*_g_, and the entanglement dynamics can be rationalized through two types of polymer–ion complex, i.e, the ion-induced intrachain conformation change and the ion-induced interchain linkage. These results point out a crucial role of the detailed monomer structures and the relative locations of the polar groups for future polymer electrolytes design.

## Supplementary Material



## Notes

(i) Comparison of Bjerrum, Keesom, and average ion separation lengths of polymer electrolytes; (ii) representative dielectric spectra of polymer electrolytes at different salt concentrations; (iii) activation plot of the secondary relaxation of polymer electrolytes; (iv) linear rheological spectra of PPO/LiTFSI electrolytes; and (v) comparison of dynamics shift factors of neat PECH and neat PPO from BDS and from linear rheology (PDF)
