# Supplementary material for: Effect of Monomer Polarity on Polymer Dynamics, Glass Transition, and Ionic Conductivity of Polyether Electrolytes
Source: Macromolecules. 2026 Jan 7;59(2):840–58. doi: 10.1021/acs.macromol.5c02520 (PMC12854761; doi:10.1021/acs.macromol.5c02520)
Supplement: Supplementary file 1 [file ma5c02520_si_001.pdf]

SUPPLEMENTARY MATERIALS

Effect of monomer polarity on polymer dynamics, glass transition, and ionic conductivity of polyether electrolytes

Soma Ahmadi, Danielle DeJonge, Niloofar Safaie, Shaylynn Crum-Dacon, Robert C. Ferrier, Jr., and Shiwang Cheng

Department of Chemical Engineering and Materials Science, Michigan State University, East Lansing, MI 48824, USA

Author Correspondence should be addressed to Shiwang Cheng at chengsh9@msu.edu

1. Comparison of the average ion separation with the Bjerrum length and the Keesom length polymer electrolytes.

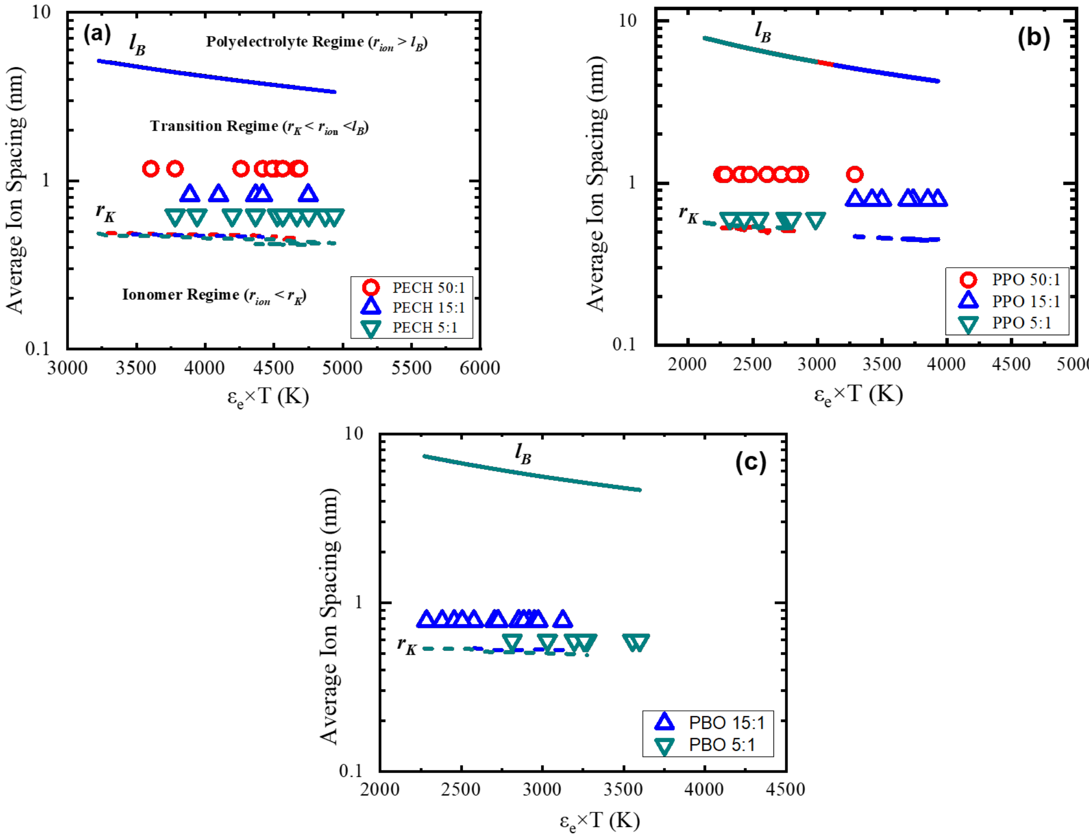

Figure S1. Bjerrum length, l_B (solid lines), Keesom length, r_K (dashed lines), and average ion spacing, r_{ion} (symbols), over $\varepsilon_e \times T$ of (a) PECH/LiTFSI electrolytes, (b) PPO/LiTFSI electrolytes, and (c) PBO/LiTFSI electrolytes.

2. Representative dielectric spectra of polymer electrolytes at different [O]:[Li] molar ratios.

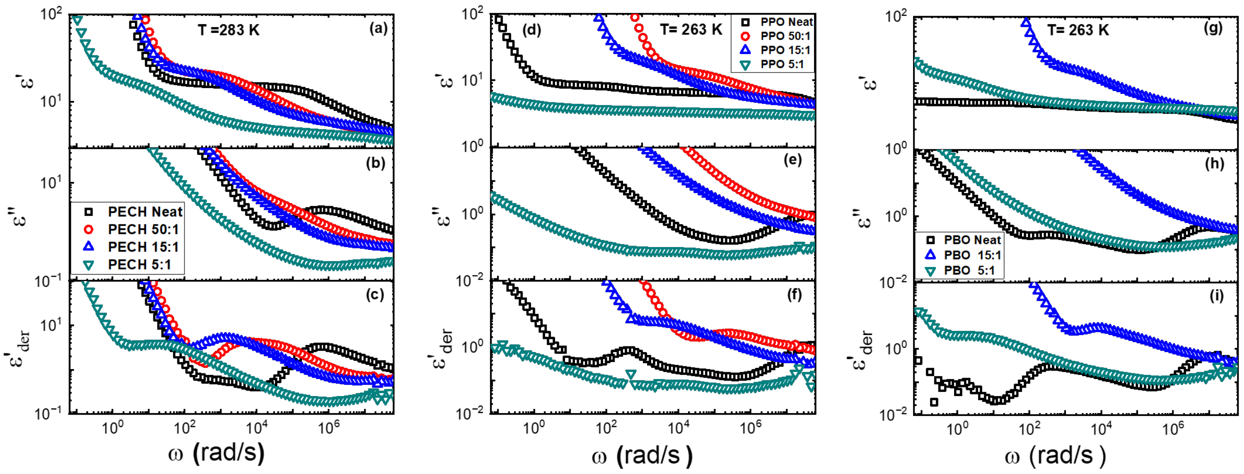

Figure S2. Dielectric spectrum, $\varepsilon'(\omega)$, $\varepsilon''(\omega)$, and $\varepsilon'_{der}(\omega)$, of (a-c) the PECH/LiTFSI electrolytes at 283 K, (d-f) the PPO/LiTFSI electrolytes at 263K, and (g-i) the PBO/LiTFSI electrolytes at 263 K. The dielectric spectra of the corresponding neat polymers (the black squares) have also been presented for comparison.

3. Activation plot of the secondary relaxation, β relaxation, of polymer electrolytes.

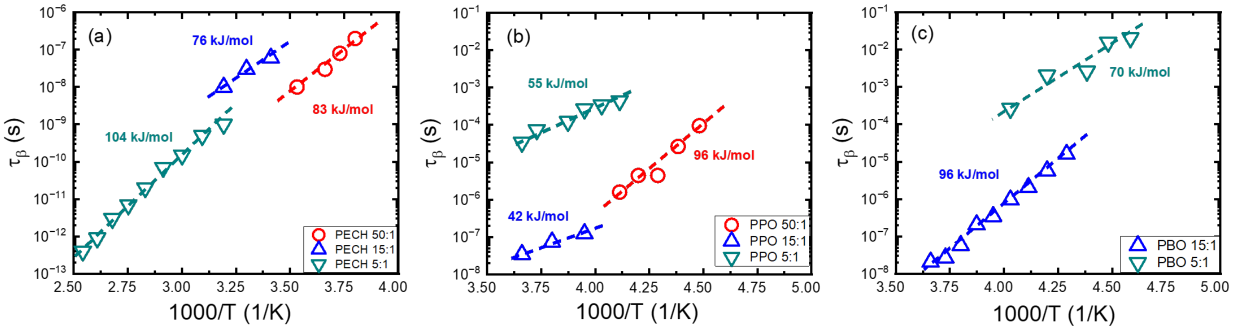

Figure S3. The secondary relaxation time τ_β for (a) PECH/LiTFSI electrolytes, (b) PPO/LiTFSI electrolytes, and (c) PBO/LiTFSI electrolytes.

4. Linear rheological spectra of PPO/LiTFSI.

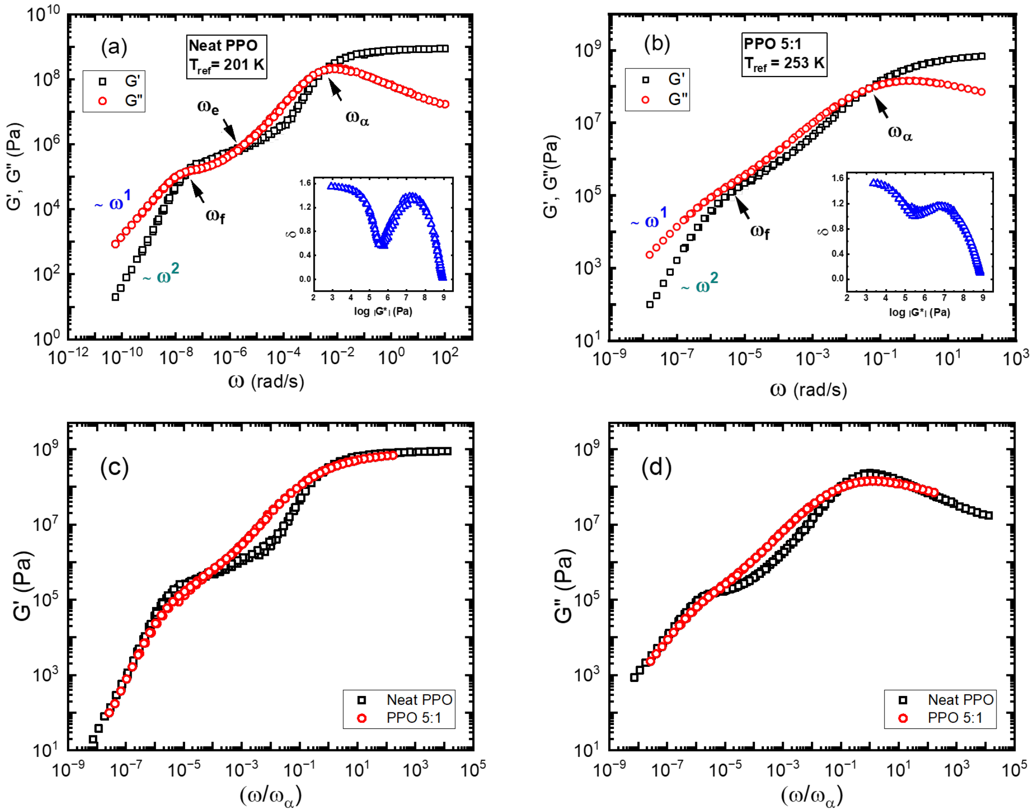

Figure S4. Linear viscoelastic master curves of (a) neat PPO at reference temperature of $T_{ref} = 201\ K$, (b) PPO/LiTFSI at [O]:[Li]=5:1 at $T_{ref} = 253\ K$. The insets in (a) and (b) are Van Gurp-Palmen plot between the phase angle δ and the $log_{10}|G^*|$ for the corresponding polymer. The comparison of the (c) the storage modulus and (d) the loss modulus of neat PPO and PPO 5:1. The x-axis is normalized by the segmental relaxation rate, ω_α.

5. Comparison of the dynamics shift factors from BDS and Rheology of neat PECH and neat PPO.

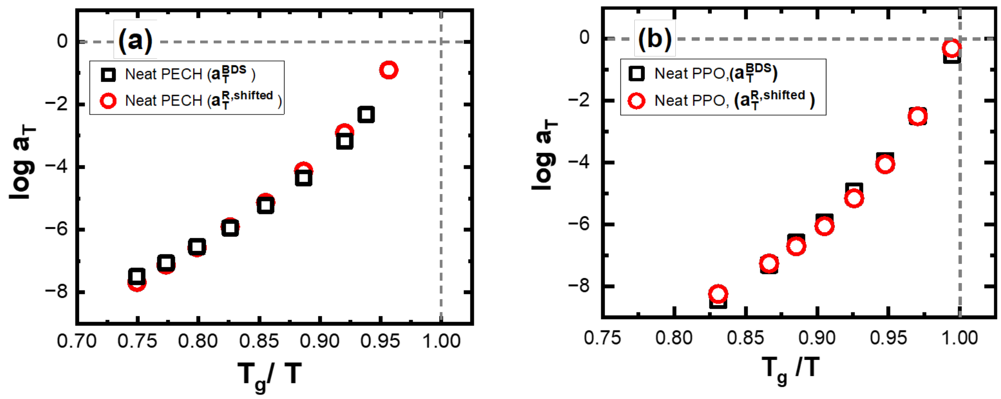

Figure S5. Comparison of the dynamics shift factor a_T^R from rheology and a_T^{BDS} from dielectric measurements of (a) neat PECH, (b) neat PPO. The a_T^R is shifted along y-axis for comparison.
